# Differentiated Instruction in Secondary Education: A Systematic Review of Research Evidence

**DOI:** 10.3389/fpsyg.2019.02366

**Published:** 2019-11-22

**Authors:** Annemieke E. Smale-Jacobse, Anna Meijer, Michelle Helms-Lorenz, Ridwan Maulana

**Affiliations:** Department of Teacher Education, University of Groningen, Groningen, Netherlands

**Keywords:** review, differentiation, differentiated instruction, adaptive teaching, ability grouping, secondary education, student performance, effectiveness

## Abstract

Differentiated instruction is a pedagogical-didactical approach that provides teachers with a starting point for meeting students' diverse learning needs. Although differentiated instruction has gained a lot of attention in practice and research, not much is known about the status of the empirical evidence and its benefits for enhancing student achievement in secondary education. The current review sets out to provide an overview of the theoretical conceptualizations of differentiated instruction as well as prior findings on its effectiveness. Then, by means of a systematic review of the literature from 2006 to 2016, empirical evidence on the effects of within-class differentiated instruction for secondary school students' academic achievement is evaluated and summarized. After a rigorous search and selection process, only 14 papers about 12 unique empirical studies on the topic were selected for review. A narrative description of the selected papers shows that differentiated instruction has been operationalized in many different ways. The selection includes studies on generic teacher trainings for differentiated instruction, ability grouping and tiering, individualization, mastery learning, heterogeneous grouping, and remediation in flipped classroom lessons. The majority of the studies show small to moderate positive effects of differentiated instruction on student achievement. Summarized effect sizes across studies range from *d* = +0.741 to +0.509 (omitting an outlier). These empirical findings give some indication of the possible benefits of differentiated instruction. However, they also point out that there are still severe knowledge gaps. More research is needed before drawing convincing conclusions regarding the effectiveness and value of different approaches to differentiated instruction for secondary school classes.

## Introduction

Differentiation is a hot-topic in education nowadays. Policy-makers and researchers urge teachers to embrace diversity and to adapt their instruction to the diverse learning needs of students in their classrooms (Schleicher, 2016; Unesco, 2017). Differentiation is a philosophy of teaching rooted in deep respect for students, acknowledgment of their differences, and the drive to help all students thrive. Such ideas imply that teachers proactively modify curricula, teaching methods, resources, learning activities, or requirements for student products to better meet students' learning needs (Tomlinson et al., 2003). When teachers deliberately plan such adaptations to facilitate students' learning and execute these adaptations during their lessons we call it differentiated instruction. A number of developments in education have boosted the need for differentiated instruction. First, contemporary classes are becoming relatively heterogeneous because of policies focused on detracking, the inclusion of students from culturally and linguistically diverse backgrounds, and inclusive education in which special education students (SEN) attend classes along with non-SEN students (Rock et al., 2008; Tomlinson, 2015). Since early stratification of students may have unintended effects on the educational opportunities of students with varying background characteristics, addressing students' learning needs by teaching adaptively within heterogeneous classrooms has been proposed as the best choice for a fair educational system (Oakes, 2008; Schütz et al., 2008; Schofield, 2010; OECD, 2012, 2018). In addition, even within relatively homogeneous classrooms, there are considerable differences between students that need attention (Wilkinson and Penney, 2014). Second, the idea that learners have different learning needs and that a one-size-fits-all approach does not suffice, is gaining momentum (Subban, 2006). Policy makers stress that all students should be supported to develop their knowledge and skills at their own level (Rock et al., 2008; Schleicher, 2016) and there is the wish to improve equity or equality among students (Unesco, 2017; Kyriakides et al., 2018). When the aim is to decrease the gap between low and high achieving students, teachers could invest most in supporting low achieving students. This is called convergent differentiation (Bosker, 2005). Alternatively, teachers may apply divergent differentiation in which they strive for equality by dividing their efforts equally across all students, allowing for variation between students in the learning goals they reach, time they use, and outcomes they produce (Bosker, 2005).

Although the concept of differentiated instruction is quite well-known, teachers find it difficult to grasp how differentiated instruction should be implemented in their classrooms (Van Casteren et al., 2017). A recent study found that teachers across different countries infrequently adapt their instruction to student characteristics (Schleicher, 2016). Struggling students may work on too difficult tasks or, conversely, high ability students may practice skills they have already mastered (Tomlinson et al., 2003). Clearly, more information about effective practices is needed. A recent review and meta-analysis of differentiated instruction practices in primary education shows that differentiated instruction has some potential for improving student outcomes, when implemented well (Deunk et al., 2018). However, these results may not generalize directly to secondary education, since the situation in which teachers teach multiple classes in secondary education is rather different in nature compared to primary education (Van Casteren et al., 2017). For secondary education, evidence for the benefits of differentiated instruction is scarce (Coubergs et al., 2013). The bulk of studies in secondary education focus on differentiation of students between classes by means of streaming or tracking (Slavin, 1990a; Schofield, 2010). Alternatively, the current study seeks to scrutinize which empirical evidence there is on the effectiveness of within-class differentiated instruction in secondary education, how studies operationalize the approach, and in which contexts the studies were performed.

## Theory and Operationalizations

### Operationalizing Differentiated Instruction in the Classroom

Theories of differentiation are bound by several guiding principles. They include a focus on essential ideas and skills in each content area, responsiveness to individual differences, integration of assessment and instruction, and ongoing adjustment of content, process, and products to meet students' learning needs (Rock et al., 2008). Differentiation typically includes pro-active and deliberate adaptations of the content, process, product, learning environment or learning time, based on the assessment of students' readiness or another relevant student characteristic such as learning preference or interest (Roy et al., 2013; Tomlinson, 2014). In Table 1, we have schematized the theoretical construct of differentiated instruction in the lesson within the broader definition of within-class differentiation.

**Table 1:**
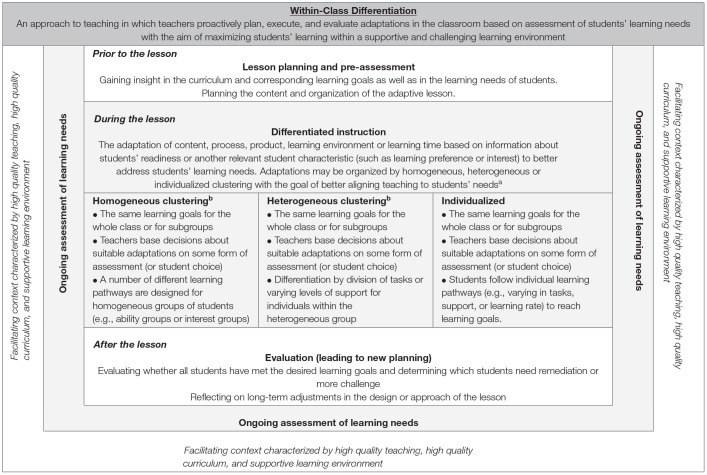
Theoretical model of within-class differentiation.

*^a^Typically teacher-directed, but ICT applications may also be used to inform or direct the differentiated instruction. ^b^Only settings in which content, process, product, environment, or learning time are purposefully adapted to the learning needs of students within or across groups are included in our model. Merely working together without any planned adaptations does not fit our definition of differentiated instruction*.

Differentiated instruction in the classroom entails two aspects. First is the *pedagogy and didactics of differentiated instruction*: which teaching practices and techniques do teachers use and what do they differentiate (McQuarrie et al., 2008; Valiande and Koutselini, 2009)? Teachers may offer students' adapted *content*, offer various options in the learning *process*, use different assessment *products*, or adapt the *learning environment* to students' learning needs (Tomlinson, 2014). Teachers may also offer certain students more *learning time* or conversely, encourage high achievers to speed up their learning process (Coubergs et al., 2013). Regarding the *process*, they may use pre-teaching or extended instruction to cater to the needs of students (Smets and Struyven, 2018), or they could adapt instructions throughout the lesson. Second, the *organizational aspect of differentiated instruction* entails the structure in which it is embedded. There are different approaches a teacher may choose (see Table 1). In macro-adaptive approaches, teachers use some form of *homogeneous clustering* to organize their differentiated instruction (Corno, 2008), including fixed or flexible grouping of students based on a common characteristic such as readiness or interest. Alternatively, teachers could use *heterogeneous grouping* to organize their differentiated instruction. Differentiation of the learning process may occur because students divide tasks within the group based on their learning preferences or abilities. Alternatively, a teacher may suggest a division of tasks or support based on assessment of learning needs (Coubergs et al., 2013). When adaptations are taken to the level at which individual students work at their own rate on their level, this is called *individualization* (Education Endowment Foundation, n.d.). The learning goals are the same, but learning trajectories are tailored to individuals' needs. Some authors include individualized approaches into the theoretical construct of differentiated instruction (Smit et al., 2011; Coubergs et al., 2013; Tomlinson, 2014), whereas others separate it from differentiated instruction (Bray and McClaskey, 2013; Roy et al., 2013).

Lastly, there are teaching models or strategies in which differentiated instruction has a central place. One well-known example is group-based *mastery learning*. In this approach, subject matter is divided into small blocks or units. For each unit, the teacher gives uniform instructions to the whole group of students. Then, a formative assessment informs the teacher which students reach the desired level of mastery of the unit (usually set at 80–90% correct). Students below this criterion receive corrective instruction in small groups, or alternatively, forms of tutoring, peer tutoring or independent practice are also possible to differentiate the learning process (Slavin, 1987). Differentiated instruction may also be embedded in other instructional approaches like peer tutoring, problem-based learning, flipped classroom models etc. (Mastropieri et al., 2006; Coubergs et al., 2013; Altemueller and Lindquist, 2017).

Immediate, unplanned adaptations to student needs, so-called “micro-adaptations” (Corno, 2008), are not included in the theoretical model in Table 1, since differentiated instruction is—by nature—planned and deliberate (Coubergs et al., 2013; Tomlinson, 2014; Keuning et al., 2017). Furthermore, we did not include the concept of “personalization” in our model since in personalized approaches students follow their own learning trajectories, pursue their own learning goals, and co-construct the learning trajectory, which makes it notably different from typical operationalizations of differentiated instruction (Bray and McClaskey, 2013; Cavanagh, 2014).

### Differentiation as a Sum of Its Parts

As noted above, differentiated instruction during the lesson is in fact only one piece of the mosaic (Tomlinson, 1999). There are a lot of other steps that are crucial for successful implementation of differentiated instruction (Keuning et al., 2017; Van Geel et al., 2019). Table 1 shows other behaviors that are related to what teachers do in the classroom. First, *continuous monitoring and (formative) assessment* and differentiated instruction are inseparable (Hall, 1992; Valiande and Koutselini, 2009; Roy et al., 2013; Tomlinson, 2014; Denessen and Douglas, 2015; Prast et al., 2015). Some teachers may be inclined to use rather one-dimensional, fixed categorizations of students based on their learning needs at some point in time (Smets and Struyven, 2018). Nevertheless, high quality differentiated instruction is based on the frequent assessment of learning needs and flexible adaptations to meet those needs. Prior to the lesson including differentiated instruction, teachers should have clear *goals* for their students, use some form of *pre-assessment*, and *plan* their adaptive instruction (Prast et al., 2015; Keuning et al., 2017; Van Geel et al., 2019). Then, teachers proceed to the actual *differentiated instruction during the lesson*. After the lesson, teachers should *evaluate* students' progress toward their goals.

Besides these steps, more general *high-quality teaching behaviors* are preconditions to create a good context for differentiated instruction (Wang et al., 1990; Tomlinson, 2014). For instance, creating a safe and stimulating learning environment in which students feel welcomed and respected is essential (Tomlinson, 2014). In addition, good classroom management may help teachers to implement differentiated instruction in an orderly manner (Maulana et al., 2015; Prast et al., 2015). In empirical studies, differentiated instruction has been found to be a separate domain of teaching, while at the same time being strongly interrelated with other high quality teaching behaviors (Van de Grift et al., 2014; Maulana et al., 2015; Van der Lans et al., 2017, 2018). In turn, high quality teaching behaviors like questioning, explaining the lesson content, or giving examples can be applied in a differentiated way, stressing that high quality teaching is both a contextual factor as a direct source of input for teachers' differentiated instruction.

### Prior Review Studies on Differentiated Instruction

Although studies on within-class differentiated instruction in secondary education are scarce, a number of reviews and meta-analyses have shed some light on the effects on student achievement. Subban (2006) discusses a number of studies showing that adapting content or processes can make learning more engaging for students than one-size-fits-all teaching, and some studies showed positive effects of differentiated instruction on student achievement. The narrative review by Tomlinson et al. (2003) revealed studies showing that students achieve better results in mixed-ability classrooms in which the teacher differentiates instruction than in homogeneous classes were a more single-size approach is used. In a recent narrative research synthesis on adaptive teaching, one study on differentiated instruction was included. The authors found positive results of different types of adaptive teaching on students' academic and non-academic outcomes in primary education (Parsons et al., 2018). In a large-scale meta-analysis by Scheerens (2016), adaptive teaching was operationalized with some relevant indicators such as using variable teaching methods, orientation toward individual learning processes, and considering students' prerequisites. In this meta-analysis, a very small effect of adaptive teaching on student achievement was found.

A number of reviews report on specific operationalizations of within-class differentiated instruction. One of the most frequently reviewed forms is *ability grouping*. In within-class ability grouping, teachers cluster students into different homogeneous groups based on their abilities or readiness. In her narrative review, Tieso (2003) summarizes that ability grouping has a potential influence on student achievement when grouping is flexible, and teachers adapt their instruction to the needs of different groups. Steenbergen-Hu et al. (2016) performed a meta-synthesis including five other meta-analyses of the effects of ability grouping in K-12 education. In their study, within-class grouping was found to have at least a small positive impact on students' academic achievement (Hedges *g* = + 0.25). In the study of Kulik (1992), who also combined results from different meta-analyses, a comparable effect size of Glass's Δ = + 0.25 in favor of within-class ability grouping was found. In the meta-analysis of Lou et al. (1996) on grouping in secondary education, within-class grouping was found to have a small positive effect (Cohen's *d* = + 0.12) on student outcomes. Substantive achievement gains were found in studies in which teachers adapted their teaching to needs of the different ability groups (Cohen's *d* = + 0.25), but not in studies in which teachers provided the same instruction for the different groups (Cohen's *d* = + 0.02). In his large meta-analysis of effects of instructional approaches on student outcomes, Hattie (2009) reported a small positive effect of within-class ability grouping on students' academic achievement (Cohen's *d* = +0.16). Conversely, Slavin (1990a) did not find significant effects of (between and within-class) ability grouping on achievement in secondary education. In a meta-synthesis of multiple meta-analyses on ability grouping—including between-class ability grouping—no overall positive effects of the approach were found (Sipe and Curlette, 1996). Some studies have found that ability grouping effects may differ for subgroups of students. For instance, Lou et al. (1996) found that low-ability students learned significantly more in heterogeneous (mixed-ability) groups, average-ability students benefitted most in homogeneous ability groups, and for high-ability students group composition made no significant difference. In primary education, Deunk et al. (2018) found a negative effect of within-class homogeneous grouping for low achieving pupils. Conversely, Steenbergen-Hu et al. (2016) concluded that high-, average-, and low-ability students all benefited equally from ability grouping. Thus, the findings on differential effects of ability grouping remain inconclusive.

Another possible approach to differentiated instruction is tiering. *Tiering* refers to using the same curriculum material for all learners, but adjusting the depth of content, the learning activity process, and/or the type of product developed by the student to students' readiness, interest or learning style (Pierce and Adams, 2005; Richards and Omdal, 2007). Teachers design a number of variations or *tiers* to a learning task, process or product, to which students are assigned based on assessed abilities. To our knowledge, there are no specific reviews of the literature or meta-analyses summarizing the effects of tiering on student achievement, but the approach is often combined with homogeneous (ability) grouping.

Alternatively, turning to *heterogeneous grouping* as an organizational structure for differentiated instruction, there is evidence that students of varying backgrounds working together may learn from each other's knowledge, from observing each other, and from commenting on each other's errors (Nokes-Malach et al., 2015). However, based on their narrative review about differentiated instruction in secondary schools, Coubergs et al. (2013) concluded that there is little known about the effectiveness of differentiated instruction in heterogeneous settings They found that guiding heterogeneous groups is challenging for teachers, and that it is difficult to address the learning needs of all students in these mixed groups.

Reviews of effectiveness of *individualized instruction* indicate small effects on student outcomes. Hattie (2009) reports a small effect of individualization on student achievement (Cohen's *d* = +0.23). In addition, in another review a wide range of effects across meta-analyses was found of individualization on academic achievement of students (from −0.07 to +0.40; Education Endowment Foundation, n.d.). Currently, mostly ICT-applications are used to individualize instruction. Review studies show that such adaptive ICT applications may considerably improve student achievement (Ma et al., 2014; Van der Kleij et al., 2015; Kulik and Fletcher, 2016; Shute and Rahimi, 2017).

Guskey and Pigott (1988) performed a meta-analysis on the effects of *group-based mastery learning* on students' academic outcomes from grade one up to college. They reported positive effects on students' academic achievement as a result of the application of group-based mastery learning for, among others, high school students (Hedges *g* = +0.48). Later on, Kulik et al. (1990) and Hattie (2009) also reported relatively large positive effects of group-based mastery learning on student achievement (ES = +0.59 and Cohen's *d* = +0.58, respectively). Low ability students were generally found to profit most from the convergent approach (Guskey and Pigott, 1988; Kulik et al., 1990). Mastery learning was among the most effective educational approaches in a meta-synthesis of multiple meta-analyses (Sipe and Curlette, 1996). However, mastery learning may be particularly valuable to train specific skills but may yield fewer positive results for more general skills as measured by standardized tests (Slavin, 1987, 1990b). Mastery learning has also been incorporated into broader interventions in secondary education such as the IMPROVE method (Mevarech and Kramarski, 1997).

Overall, from previous review studies we can draw the conclusion that there is some evidence that differentiated instruction has potential power to affect students' academic achievement positively with small to medium effects. However, the evidence is limited and heterogeneous in nature. The effectiveness of some approaches to differentiated instruction, such as ability grouping, has been reviewed extensively, while other approaches have received less attention. Furthermore, most studies were executed some time ago and were executed in the context of primary education, while only few studies focus specifically on secondary education.

### Contextual and Personal Factors Influencing Differentiated Instruction

When analyzing the effectiveness of differentiated instruction, it is important to acknowledge that classroom processes do not occur in a vacuum. Both internal and external sources determine whether teachers will succeed in developing complex teaching skills (Clarke and Hollingsworth, 2002). In the case of differentiated instruction, teacher-level variables like education, professional development and personal characteristics like knowledge, attitudes, beliefs, values and self-efficacy may influence their behavior (Tomlinson, 1995; Tomlinson et al., 2003; Kiley, 2011; De Jager, 2013; Parsons et al., 2013; Dixon et al., 2014; De Neve and Devos, 2016; Suprayogi et al., 2017; Stollman, 2018). Teachers need thorough content knowledge and a broad range of pedagogical and didactic skills to plan and execute differentiated instruction (Van Casteren et al., 2017). At the classroom level, diversity of the student population (De Neve and Devos, 2016) and class-size (Blatchford et al., 2011; Suprayogi et al., 2017; Stollman, 2018) influence interactions between teachers and their students. Moreover, school characteristics matter. For instance, a school principal's support can influence implementation of differentiated instruction (Hertberg-Davis and Brighton, 2006). Additionally, structural organizational conditions, such as time and resources available for professional development, and cultural organizational conditions such as the learning environment, support from the school board, and a professional culture of collaboration may influence teaching (Imants and Van Veen, 2010; Stollman, 2018). Teachers have reported that preparation time is a crucial factor determining the implementation of differentiated instruction (De Jager, 2013; Van Casteren et al., 2017). Moreover, collaboration is key; a high pedagogical team culture influences both the learning climate and the implementation of differentiated instruction (Smit and Humpert, 2012; Stollman, 2018). Lastly, country level requirements and (assessment) policies that stress differentiated instruction may influence implementation (Mills et al., 2014).

## Research Questions

Researchers and teachers lack a systematic overview of the current empirical evidence for different approaches to within-class differentiated instruction in secondary education. Therefore, we aim to (1) give an overview of the empirical literature on effects of differentiated instruction on student achievement in secondary education, and (2) consider the degree to which contextual and personal factors inhibit or enhance the effects of within-class differentiated instruction.

Our study is guided by the following research questions:

RQ1. What is the research base regarding the effects of within-class differentiated instruction on students' academic achievement in secondary education?
RQ2. How are the selected approaches to differentiated instruction operationalized?
RQ3. What are the overall effects of differentiated instruction on students' academic achievement?
RQ4. Which contextual and personal factors inhibit or enhance the effects of differentiated instruction on student achievement?

Based on previous research, we hypothesize to find literature on multiple possible approaches to differentiated instruction in the classroom. Probably, there will be more evidence for some operationalizations (like ability grouping) than for others. Overall, we hypothesize that differentiated instruction will have a small to medium positive effect on students' academic achievement. Several contextual and personal factors may affect the implementation. In this review, we will include information about relevant contextual and personal variables—when provided—into the interpretation of the literature.

## Methods

### Study Design

In order to provide a systematic overview of the literature on within-class differentiated instruction, a best evidence synthesis (Slavin, 1986, 1995; Best Evidence Encyclopedia, n.d.) was applied. This was done by a-priori defining consistent, transparent standards to identify relevant studies about within-class differentiated instruction. Each selected study is discussed in some detail and results are evaluated. In case enough papers are found that are comparable, findings can be pooled across studies. The best-evidence strategy is particularly suitable for topics—such as differentiated instruction—for which the body of literature is expected to be rather small and diverse. In such cases, it is important to learn as much as possible from each study, not just to average quantitative outcomes and study characteristics (compare Slavin and Cheung, 2005). In a recent review study on differentiated instruction in primary schools, the best evidence synthesis approach was used as well (Deunk et al., 2018). In this study, the authors mentioned the benefits of selecting studies using strict pre-defined criteria (to avoid a garbage in-garbage-out effect). Moreover, combining a meta-analysis with relatively extended descriptions of the included studies in order to make the information more fine-grained was found to improve the interpretability of the results.

### Working Definition of Differentiated Instruction

To select relevant studies for our review, we used the following working definition of differentiated instruction: Differentiated teaching in the classroom consisting of planned adaptations in process, learning time, content, product or learning environment for groups of students or individual students. Adaptations can be based on achievement/readiness or another relevant student characteristic (such as prior knowledge, learning preferences, and interest) with the goal of meeting students' learning needs.

Adaptations that are merely organizational, such as placing students in homogeneous groups without adapting the teaching to relevant inter-learner differences, were excluded. Interventions using approaches like peer tutoring, project-based learning and other types of collaborative leaning were eligible, but only when planned differentiated instruction was applied based on relevant student characteristics (e.g., by assigning specific roles based on students' abilities). Beyond the scope of this review were studies on differentiated instruction outside the classroom such as between-class differentiation (streaming or tracking), tutoring outside the classroom, or stratification of students between schools.

### Search Strategy

The studies for our best evidence synthesis were identified in a number of steps. First, we performed a systematic search in the online databases ERIC, PsycINFO, and Web of Science (SSCI). Following the guidelines of Petticrew and Roberts (2006), a set of keywords referring to the intervention (differentiation combined with keywords referring to instruction), the population (secondary education) and the outcomes of interest (academic outcomes) were used. We limited the findings to studies published between 2006 and 2016 that were published in academic journals. Although this first search yielded relevant studies, it failed to identify a number of important studies on differentiated instruction practices known from the literature. This was because search terms like “differentiation” and “adaptive” were not used in all relevant studies. Some authors used more specific terms such as ability grouping, tiered lessons, flexible grouping and mastery learning. Therefore, an additional search was performed in ERIC and PsycINFO with more specific keywords associated with differentiated instruction. We added keywords referring to various homogeneous or heterogeneous clustering approaches, to mastery learning approaches, or to convergent or divergent approaches (see Appendix A for the full search string)^1^.

Additional to this protocol-driven approach, we used more informal approaches to trace relevant studies. We cross-referenced the selected papers and recent review studies on related topics, used personal knowledge about relevant papers, and consulted experts in the field. We only used newly identified papers in case they were from journals indexed in the online databases Ebscohost, Web of Science, or Scopus to avoid selecting predatory journal outputs.

### Selection of Papers

The identified papers were screened in pre-designed Excel sheets in two stages. First, two independent coders applied a set of inclusion criteria (criteria 1–8) to all papers based on title, abstract, and keywords. The papers that met the following conditions were reviewed in full text: (1) one or both of the coders judged the paper to be included for full text review based on the inclusion criteria using the title, abstract, and keywords, or (2) the study fulfilled some of the inclusion criteria but not all criteria could be discerned clearly from the title, abstract or keywords. Second, in a full text review, two coders applied the inclusion criteria again after reading the full paper. If a study met the basic criteria 1–8, additional methodological criteria (9–13) were checked in order to make the final selection. To assure the quality of the coding process, full-text coding of both coders was compared. Differences between coders about whether the study met certain inclusion criteria were resolved by discussion and consensus. The dual coding process by two reviewers was used since this substantially increases the chance that eligible studies are rightfully included (Edwards et al., 2002). Only studies that met all 13 inclusion criteria were included in the review.

### Inclusion Criteria

The following inclusion criteria were used to select the relevant papers. These criteria were based on a prior review study on differentiated instruction in primary education (Deunk et al., 2018) and the best evidence studies by Slavin and colleagues (Slavin and Cheung, 2005; Slavin et al., 2008, 2009; Slavin, 2013; Cheung et al., 2017).

1. *Within-class differentiated instruction:* The study is about the effect of within-class differentiated instruction, as defined in our study (see section Working Definition of Differentiated Instruction).
2. *Practicality*: The differentiated instruction approach is practical for teachers (Janssen et al., 2015). Teachers must be able to apply this intervention themselves in a regular classroom. In addition, the intervention is time- and cost-effective, meaning that it should not take excessive training or coaching nor use of external teachers in the classroom to implement the approach. Interventions in which ICT applications are used to support the teachers' instruction and can be controlled by the teacher (e.g., in blended learning environments in which teachers make use of on-line tools or PowerPoint) could be included. However, studies on the effects of fully computerized adaptive programs (e.g., with adaptive feedback or intelligent tutors) or differentiation approaches for which an external teacher (or tutor) is needed (such as pullout interventions) were excluded.
3. *Study type:* Students in a differentiated instruction intervention condition are compared to those in a control condition in which students are taught using standard practice (“business as usual”), or to an alternative intervention (compare Slavin et al., 2008, 2009; Slavin, 2013; Cheung et al., 2017; Deunk et al., 2018). The design could be truly randomized or quasi-experimental or matched (the control condition could be a group of other students in a between-group design, or students could be their own control group in a within-groups design)^2^. Additionally, large-scale survey designs in which within-class differentiated instruction is retrospectively linked to academic outcomes were eligible for inclusion (compare Deunk et al., 2018). Surveys have increasingly included been used in reviews of effectiveness, although one must keep in mind that no finding from a survey is definitive (Petticrew and Roberts, 2006).
4. *Quantitative empirical study*: The study contains quantitative empirical data of at least 15 students per experimental group (compare Slavin et al., 2008, 2009; Slavin, 2013; Cheung et al., 2017; Deunk et al., 2018). Other studies such as qualitative studies, case studies with fewer than 15 students, or theoretical or descriptive studies were excluded.
5. *Secondary education:* The study was executed in secondary education. For example, in middle schools, high schools, vocational schools, sixth-form schools or comparable levels of education for students from an age of about 11 or 12 years onwards. In some contexts, secondary schools could include grades as low as five, but they usually start with sixth or seventh grades (compare Slavin, 1990a).
6. *Mainstream education*: The study was performed in a mainstream school setting (in a regular school, during school hours). Studies that were performed in non-school settings (e.g., in a laboratory or the workplace) or in an alternate school setting (e.g., an on-line course, a summer school, a special needs school) were excluded.
7. *Academic achievement*: Academic achievement of students is reported as a quantitative dependent variable, such as mathematics skills, language comprehension, or knowledge of history.
8. *Language*: The paper is written in English or Dutch (all authors master these languages), but the actual studies could be performed in any country.

Additional inclusion criteria used in the full-text review:

1. *Differentiated instruction purpose:* The study is about differentiated instruction with the aim of addressing cognitive differences (e.g., readiness, achievement level, intelligence) or differences in motivation / interest or learning profiles (Tomlinson et al., 2003). Studies in which adaptions were made based on other factors such as culture (“culturally responsive teaching”) or physical or mental disabilities are beyond the scope of this review.
2. *Implementation*: The intervention is (at least partly) implemented. If this was not specifically reported, implementation was assumed.
3. *Outcome measurement:* The dependent variables/outcome measures include quantitative measures of achievement. Experimenter-made measures were accepted if they were comprehensive and fair to the both groups; no treatment-inherent measures were included (Slavin and Madden, 2011).
4. *Effect sizes*: The paper provides enough information to calculate or extract effect sizes about the effectiveness of the differentiated instruction approach.
5. *Comparability*: Pretest information is provided (unless random assignments of at least 30 units was used and there were no indications of initial inequality). Studies with pretest differences of more than 50% of a standard deviation were excluded because—even with analyses of covariance—large pretest differences cannot be adequately adjusted for (Slavin et al., 2009; Slavin, 2013; Cheung et al., 2017; compare Deunk et al., 2018).

### Data Extraction

After the final selection of papers based on the criteria above, relevant information was extracted from the papers and coded by two independent reviewers in a pre-designed Excel sheet (see Appendix B). Discrepancies between the extractions of both reviewers were discussed until consensus was reached. Missing information regarding the methodology or results was requested from the authors by e-mail (although only few responses were received). The content coding was used (additional to the full texts) to inform the literature synthesis and to extract data for the calculation of effect sizes.

### Data Analysis

We transformed all outcomes on student achievement from the selected papers to Cohen's *d*, which is the standardized mean difference between groups (Petticrew and Roberts, 2006; Borenstein et al., 2009). To do so, the program Comprehensive Meta-Analysis (CMA) version 2 was used (Borenstein et al., 2009). Effect sizes were calculated using a random effects model since we have no reason to assume that the studies are “identical” in the sense that the true effect size is exactly the same in all studies (Borenstein et al., 2010). Methods of calculating effects using different types of data are described in Borenstein et al. (2009) and Lyons (2003). When outcomes were reported in multiple formats in the paper, we chose the means and standard deviations to come to transparent and comparable outcomes. The effects were standardized using post-score standard deviations for measures where this was needed. For some outcome formats, CMA requires the user to insert a pre-post correlation. Since none of the selected papers provided this number, we assumed a correlation of 0.80 in the analyses since it is reasonable to assume such a pre- post correlation in studies in secondary education (Swanson and Lussier, 2001; Cole et al., 2011). This correlation does not affect the Cohen's *d* statistic but has impact on its variance component. For the papers in which multiple outcome measures were reported, we used the means of the different measures. In case only subgroup means (of subgroups within classes of schools) were reported, we combined the outcomes of the subgroups with study as the unit of analysis to calculate a combined effect (Borenstein et al., 2009). For one study in which the intervention was executed in separate schools differing in implementation and findings, we have included the schools in the analyses separately (using schools in which the intervention took place as the unit of analysis).

## Results

### Search Results

Our search led to 1,365 hits from the online databases ERIC, PsycINFO and Web of Science and 34 cross-referenced papers. Excluding duplicates, 1,029 papers were reviewed. See Appendix C for a flow-chart of the selection process. In total, 14 papers met the eligibility criteria for inclusion. Papers reporting on the same project and outcomes were taken together as one study. The papers by Altintas and Özdemir (2015a,b) report on the same project. The same applies to two other papers as well (Vogt and Rogalla, 2009; Bruhwiler and Blatchford, 2011). Thus, in the end, 12 unique studies were included in our review and meta-analysis leading to 15 effects in total (since for one study the four different schools in which the intervention was executed were taken as the unit of analysis).

### Study Characteristics

In Table 2, the characteristics and individual effects of the studies included in our review are summarized. The selection of studies includes eight quasi-experimental studies in which classes were randomly allocated to a control or experimental condition (Mastropieri et al., 2006; Richards and Omdal, 2007; Huber et al., 2009; Vogt and Rogalla, 2009; Little et al., 2014; Altintas and Özdemir, 2015a,b; Bal, 2016; Bhagat et al., 2016), three studies in which schools were randomly allocated to conditions (Wambugu and Changeiywo, 2008; Mitee and Obaitan, 2015; Bikić et al., 2016), and one survey-study (Smit and Humpert, 2012). These studies covered a wide range of academic subjects, including science, mathematics and reading. In terms of the number of participating students, six studies were small-scale studies (*N* < 250) and six were large-scale studies (*N* > 250). However, note that all experiments had nested designs. Only the studies of Little et al. (2014) and Vogt and Rogalla (2009) have at least 15 cases in each experimental condition at the level of randomization. Four studies were performed in the United States of America, five in Europe, one in Taiwan, and two in Africa. All studies were performed in secondary education, but the Vogt and Rogalla study represents a combined sample of primary- and secondary education students.

**Table 2:** Summary of contents of the selected papers and the effects of the individual studies on student achievement.

**Paper**	**Country**	**Sample (analyses)**	**What is differentiated and how?**	**Approach**	**Subject**	**Assessment of learning needs**	**Teacher support**	**Duration and intensity of intervention**	**Study design**	**Control condition**	**Effect on student performance (Cohens' *d*) and 95% CI**	**Relevant context characteris-tics**
Altintas and Özdemir (2015a,b)	Turkey	Grade 6: 32 students contr., 28 students exp.Grade 7: 42 students contr., 42 students exp.(age 13–14)	Adaptations were made in *content, process, product, and learning environment*. The student inventory was used to determine the students' preferred project topics, select teaching strategies, and relevant factors for motivating students. Upper-grade objectives were selected for enrichment.	A *project-based* interdisciplinary approach in which students were asked to select project topics by considering their dominant intelligences, the newly developed differentiation approach, creativity strategies, and the subject objectives.	Math	*Learning profile* based on a multiple intelligences inventory for students	The researchers developed different project topics suitable to students' skills and interests. Teachers were informed about the activities that would be conducted in meetings.	Not clearly reported. Probably 42 weeks: six math topics, 7 weeks per topic.	Quasi-experi-mental, pretest posttest.	National educational curriculum activities from the Purdue Model.	Mathematics achievement test^A^ (researcher-made) Grade 6 *d* = +6.242^**^ [5.015 to 7.468] Grade 7 *d* = +3.893^**^ [3.165 to 4.621] *Combined d* = +4.504^**^ [3.879 to 5.130]	Gifted classes and non-gifted classes were included.
Bal (2016)	Turkey	Grade 6: 24 students contr., 33 students exp.	Differentiation of *content* and *instruction*. The authors state “Lesson plans and activities were formed according to the students' learning styles and readiness levels”	A *tiering* approach in which students are assigned to different materials/activities by the teacher based on their reported learning styles and a pretest (two tiers: low readiness and medium readiness).	Math (algebra)	An algebra pre-test was used to determine *readiness* (less than half correct = low readiness, the rest medium readiness) and an inventory was used to determine *learning styles* (kinesthetic, visual, affective).	The researcher prepared lesson plans, activities, worksheets, and all materials and observed most tiered lessons. The lessons for both the experiment and control groups were conducted by a mathematics postgraduate student teacher	4 weeks (16 lesson hours)	Quasi-experi-mental, pretest posttest, follow-up	Business as usual (taught by the same teacher)	Algebra test^B^ (researcher-made) *d* = + 1.085^**^ [0.479 to 1.692]	Lessons taught by a student teacher
Bhagat et al. (2016)	Taiwan	41 students contr. (low 8, average 19, high 14), 41 students exp.(age 14–15)	Since remediation followed the instructional video's it seems that there was differentiation of *process* (remedial instruction) for weak students. It is unclear whether for the rest of the students working in groups there was also any planned differentiation.	Students in the *flipped classroom* condition watched instructional videos at home. During the lesson, students worked *collaboratively* to discuss problems and students who needed remediation were given *extra instruction*.	Math (trigonometry)	Not reported	Not reported	6 weeks	Quasi-experimental, pretest posttest	Business as usual; 30–40 min lecture and discussion, remaining time (of the total 50 min) problem solving)	Mathematics achievement test (multiple choice, researcher -made)^C^ Low ability *d* = +0.826 [−0.195 to 1.846] Average ability *d* = + 0.361 [−0.301 to 1.031] High ability *d* = + 0.176 [−0.532 to 0.885] *Combined* *d* = 0.376 [−0.064 to 0.815]	More males than females in the classes
Bikić et al. (2016)	Bosnia and Herzegovina	Third grade of high school: 77 students contr. (32 low, 31 average, 14 high), 88 students exp.(mean age 17)	The *content* (learning task) and *product* (type of answer students have to give) were adapted for three different groups.	*Ability grouping* with a below-average group (initial test below 40%), an average group (between 40 and 75% correct) and an above average group (above 75% correct) who work on a *problem-based learning* task.	Math	*Readiness*, assessed by a math test.	Teachers received “clear instructions and obtained activity contents.”	16 lessons	Quasi-experi-mental, pretest posttest.	Business as usual.	Mathematics achievement test^D^ (researcher-made) Low ability *d* = +0.873^**^ [0.393–1.354] Average ability *d* = +0.893^**^ [0.382–1.403] High ability d = 0.227 [−0.547 to 1.000] *Overall d =* +0.539^**^ [0.228–0.851]	The sample consists of students with technical orientation.
Huber et al. (2009)	USA	Grade 6–8: 192 students contr., 978 students exp.(age 11–15; 92% 11–13)	Not clearly reported what teachers adapted during the intervention lessons, but in the training teachers were presented with different ways to adapt *content, process, product, environment, or learning time*.	Not clearly reported. Teachers were taught to recognize students' unique *learning styles* in the context of the Prevention through Alternative Learning Styles (PALS) program and adapt the messages to these learning styles.	Alcohol, tobacco and other drug (ATOD) prevention	Students determined their preferred *learning profile* (not reported how).	In 24 classes, a PALS project staff member teaches the lessons, in the other 16 classes, the teacher teaches the lessons and receives support in five short booster sessions. The four teachers who taught the 16 classes themselves participated in a day-long training session about modifying the ways that information is presented and how instruction is given.	5 topic areas presented in 10 lessons	Quasi-experi-mental, pretest posttest	Business as usual: the schools' traditional prevention program.	Alcohol, tobacco and other drug survey^E^ (researcher-made) *d* = +1.374^**^ [1.209 to 1.538]	Sample largely Caucasian: 65%, 17% African American, 12% multi-racial, or other.
Little et al. (2014)	USA	Grade 6–8: 832/830 students contr., 1179/1198 students exp. (depending on the test used).	The *content* (books) was adapted to students' interest and level by choice. During individualized conferences teachers adapted *instruction* to students' needs.	*Individualized approach:* students self-selected challenging books in areas of interest. While studentsread independently, teachers conducted individualized conferences during whichthey assessed the challenge level of a student's book, provided instruction in reading skills and strategies as appropriate for each student, and asked and discussed higher level questions.	Reading	*Readiness* and *interest*: students self-selected challenging books and in the individualized conferences teachers assessed the needs by talking with the student.	Treatment group teachers participated in a day-long session providing an overview, modeling, and practice with the SEM-R. Additional professional development included a follow-up group session as well as ongoing classroom support from members of the project staff (approximately once every 2–3 weeks).	7 months; 40–45 min per day or 3 h per week.	Quasi-experi-mental multi-site cluster randomized design, pretest posttest.	Business as usual: regular reading instruction, which included textbook instruction, group or class novel studies, and other whole or small-group approaches.	Reading comprehension test^F^ (standardized) and Fluency test East MS *Combined d* = +0.030 [−0.138 to 0.198] North MS *Combined d* = +0.007 [−0.193 to 0.207] South MS *Combined d* = +0.266^*^ [0.015 to 0.517] West MS *Combined d* = +0.135 [−0.001 to 0.272]	Sample with high percentages of students from low-income backgrounds. Approx. 60% of students or fewer achieved passing levels on state reading tests.
Mastropieri et al. (2006)	USA	Grade 8: in total 213 students (44 classified with disabilities)(mean age about 13.5)	The *content* (assignments) and *instruction* (support in the materials) were adapted to students' abilities in three tiers.	A combination of *collaborative learning* (peer tutoring) with *tiered* content. Students worked together in groups of two or three. Students requiring assistance were paired with higher achieving partners. In the groups, students worked with materials that were differentiated based on their relative abilities.	Science	*Readiness*: teachers selected the starting level of materials (i.e., low, middle, or high) for the dyads.	The researchers developed three levels of materials for each area.	12 weeks including pretesting, teacher and student training,post testing, and surveys.	Quasi-experi-mental randomized field trial.	Business as usual: traditional instruction consisted of teacher lecture, class notes, laboratory-like class activities, and supplementary textbook materials.	Science achievement^G^ Unit test *d* = +0.466^**^ [0.194 to 0.738] High stakes end of year test *d* = +0.306^*^ [0.036 to 0.576] *Combined* *d* = +0.386^**^ [0.115 to 0.657]	The 13 inclusive classes were taught by 4 general education teachers and 4 special education teachers. All teachers were female with a mean of 2.9 years in their current position and a mean total number of 4.9 years of teaching.
Mitee and Obaitan (2015)	Nigeria	Senior secondary school (grade/age not reported): 194 students contr., 207 students exp.	No information about the specifics of the intervention is provided in the methods section. The authors do state the following in the introduction: “The students that did not gain mastery are given *corrective instructio*n based on the identified areas of difficulties from the results of the formative test and the test is administered to them again. The corrective instruction could be done through reteaching, peer tutoring, homework, small group discussion, etc.”	Group-based *mastery learning*.	Science (chemistry)	Not clearly reported. But based on the introduction we deduce that formative tests were used, implying selection based on *readiness*.	Not reported	2 weeks	Quasi-experimental, pretest posttest.	Business as usual.	Chemistry achievement test (not reported who developed the test)^H^ *d* = +1.461^**^ [1.241 to 1.682]	Not reported.
Richards and Omdal (2007)	USA	High school freshman: 143 students contr. (low 22, mid 95, high 31), 150 students exp. (low 31, mid 91, high 28)	The curriculum *content*, the *process* method(s) (and *learning time*), or the type/ complexity/depth of *product*.	*Homogeneous clustering*: *tiered* instruction and *ability grouping*.	Science (astronomy/ Newtonian physics)	*Readiness*: assessed by a pretest.	Teachers received professional development in tiered instruction 4 months before the intervention. Then, workshops were conducted for the experimental teachers to discuss the elements and methods of differentiated instruction. The researcher met with teachers twice-weekly and with individual teachers as needed for information and support. One of the researchers produced the instructional materials for the study.	4 weeks of instruction	Quasi-experimental, pretest posttest.	All learners in the control classrooms used the activities and labs designed for midrange learners in the treatment group. Control teachers may have differentia-ted to some degree, but not consistent.	Science achievement test^I^ (researcher-made) Low background knowledge *d* = +1.057^**^ [0.474 to 1.639] Midrange background knowledge *d* = +0.222 [−0.067 to 0.510] High background knowledge *d* = +0.077 [−0.434 to 0.588) *Overall d* = +0.284^*^ [0.054 to 0.514]	The student population was highly mobile and students entering high school had varying skill levels and past learning experiences.
Smit and Humpert (2012)	Switzerland	Academic outcomes were reportedfrom 351 secondary school students; 162 teachers (133 from secondary schools) participated in the study	Most teachers reported to adapt the *content, process and learning time* by providing individual tasks (tiered assignments), adapting the number of tasks or providing more time to work on tasks. These practices were not performed on a daily basis, but were implemented on an occasional basis as add-ons to regular instruction	Different approaches such as: *Individualizing, tiering, peer-tutoring*	Student outcomes in language and math	Not reported	N/A	N/A	Survey-design	N/A	Electronic achievement test German^J^ (standardized) *d* = −0.092 [−0.287 to 0.095] Electronic achievement test mathematics (standardized) *d* = −0.085 [−0.271 to 0.102] *Combined d* = −0.088 [−0.275 to 0.098]	School in the sample were small. The number of students in the secondary schools ranged from 14 to 132 with a mean of 60 students. The teachers' mean duration of service was 17.3 years (SD = 11.5, with a range of 1–43 years)
Vogt and Rogalla (2009) the project is also reported in Bruhwiler and Blatchford (2011)	Switzerland	Primary and secondary school combined: 299 students contr., 591 students exp.	Within the concept of adaptive teaching competency, it is assumed that *a variety of teaching methods* are used. Questions teacher may address, for instance: in what ways will students make their thinking and understanding public (product), how do you plan to assist those students who you predict will have difficulties and what extensions or challenges will you provide for students who are ready for them (product).	Not specified (*different approaches* are possible).	Science (biology)	*Readiness* and *interest*: the teacher should meet students' diverse skills and interests. Not specified how these are determined.	A 2-day seminar on “Adaptive Teaching Competency” and nine 3-h sessions of content-focused coaching whereby a coach visits the teachers in their classroom.	8 lessons	Quasi-experimental, pretest posttest	Not reported	Scientific literacy test (researcher-made)^K^ *d* = +0.133 [−0.006 to 0.272]	Teachers volunteered to participate. Years of teaching experience ranged from 2 to 35 years, with an average of 15 years.
Wambugu and Changeiywo (2008)	Kenya	Form 2 students (the second stage of secondary education): 81 students control (37 students with pretest), 80 students exp. (of which 35 students have a pretest.	No information about the specifics of the intervention is provided in the methods section. In the introduction the authors do note the important role of *supplementary instruction* and *corrective activities* of small units of the subject matter which seems to imply adaptation of *process and maybe content* to help students gain mastery.	*Mastery learning*.	Science (physics)	*Readiness*: level of mastery on diagnostic tests	A manual was constructed for the teachers in the mastery learning condition. These teachers were trained by the researcher on how to use the manual. They practiced with the mastery learning approach for 1 week before the start of the intervention.	3 weeks	Quasi-experimental, pretest posttest	Business as usual	Science test^L^ (researcher-made) *d* = +1.322^**^ [0.948 to 1.695]	Not reported.

* *significant at the 0.05 level*,

** *significant at the 0.01 level*.

A *In these two papers, identical main results are presented, therefore we treat the papers as one study in the table. We have used the results of the non-gifted sample only, since the gifted students were in separate classes which does not fit our selection criteria. Note that the it seems that the pretest-scores of the grade 7 students were non-normally distributed (since the authors use a non-parametric test) and also the pre-test scores are not provided. The combined effect of these two subgroups was calculated in CMA (using study as the unit of analysis)*.

B *For our analyses, we used information from the ANCOVA in Table 4 of the paper of Bal (2016) to calculate a correlation and used this in CMA to calculate Cohen's d. Note that the values are from an ANCOVA, implying they may be positively biased*.

C *For our analyses, we used means of the subgroups in the classes from Table 3 from the paper of Bhagat et al. (2016) to calculate an overall effect (using study as the unit of analysis). The pretest and posttest consisted of the same items but in a different order. It is remarkable that in most subgroups, students performed worst on the posttest than on the pretest, suggesting that on average the learning effect of answering the same items twice was limited*.

D *For our analyses, we used the overall means and standard deviations of control and experimental group from Table 1 from the paper of Bikić et al. (2016). Subgroup results were also calculated. Do note however that the subgroups are small and in some cases differ considerably on the pretest which may have biased the results*.

E *This paper reports on two studies: a quasi-experimental study with a control group and a within-group repeated measures study. We will use the results (means) of the quasi-experimental study because of the more rigorous design*.

F *Since the authors note that the implementation and the treatment effects were found to differ between schools “it is inappropriate to infer an overall treatment effect from these results” (p. 394), we have included the separate schools as rows in our analysis (thus using schools as the unit of analysis). Within schools, we used the effects reported for each outcome per school reported in Table 5 of the Little et al. (2014) paper and calculated the mean effect across outcomes*.

G *For both outcomes, we used the adjusted means from Table 2 in the paper of Mastropieri et al. (2006) to calculate a mean difference and corresponding common SD. In CMA the overall effect was calculated using the mean of the selected outcomes*.

H *Calculated in CMA using pretest and posttest means*.

I *For the analyses, we used the overall means reported in Tables 7 and 8 of the Richards and Omdal (2007) paper*.

J *We used numbers from re-analyses containing only the secondary school students shared with us by the first author. We used the t-value (division of the estimate by its standard error) to calculate the effect sizes in CMA. The combined effect was calculated in CMA using the mean of both outcomes*.

K *For our analyses, we used the overall means for the combined sample of primary and secondary students to calculate an effect size in CMA. The data reports on the same study as the Bruhwiler and Blatchford (2011) paper, but we use the results from the Vogt paper because the study design better matches our research question*.

L *We calculated Cohen's d using the F-value from the ANOVA in Table 2 of the Wambugu and Changeiywo (2008) paper with primary education scores as covariate using the formula $r=\;\surd \frac{F}{F+df_{\left(e\right)}}$. Please do note that of the 161 students in the study, only 35 from the experimental group and 37 from the control group had a formal pretest due to the research design*.

### Literature Synthesis

To further reflect on the findings from the selected studies in respect to our research questions, we will give a more detailed description of the study designs, implementations and findings here.

#### Studies on Generic Approaches to Differentiated Instruction

Although adaptive teaching does not necessarily include differentiated instruction, we found two quasi-experimental studies on adaptive teaching that (to some extent) matched our definition of differentiated instruction. In the large-scale study by Vogt and Rogalla (2009), teachers were trained in adaptive teaching competency to improve their teaching and, in turn, to maximize students' learning. In the project “Adaptive Teaching Competency,” that was also included in the paper of Bruhwiler and Blatchford (2011), adaptive teaching was characterized as including: sufficient subject knowledge, taking the diverse pre-conditions and learning processes of students into account, using various effective teaching methods for the whole group, differentiating for students' varying learning needs, supporting students in the regulation of learning processes, and using effective classroom management. In the project, teachers learned to focus on both adaptive planning prior to the lesson, as well as making adaptations during the lesson. Teachers of 27 primary school classes and 23 secondary school classes with 623 students were recruited to learn more about adaptive teaching. They participated in a 2-day workshop, received several coaching sessions in the classroom and used the adaptive teaching framework in their classes for eight science lessons. After the intervention, it was measured—among others—whether teachers differentiated to meet students' diverse skills and interests. After the intervention, teachers' competency in planning adaptive lessons significantly increased but their “Adaptive Implementation” did not change much. Unfortunately, in the coaching sessions, teachers often did not discuss about issues of adapting to the diversity of students' skills and their pre-existing knowledge. The results of students in the experimental classes were compared to those of 299 control students. The authors reported that the secondary students in the experimental group outperformed their counterparts in control classrooms on a science achievement test after the intervention. However, since we only had access to the means of the combined sample in primary and secondary education we used the combined sample results. Our calculation based on these means shows a small non-significant intervention effect of *d* = +0.133 (see Table 2). The authors argue that more coaching may be needed to foster the implementation of adaptive teaching in the classroom, although it would decrease the cost-effectiveness of the approach.

In the study by Huber et al. (2009), teachers learned about adaptive teaching in a workshop, and were asked to incorporate it into their lessons. The intervention was the Prevention through Alternative Learning Styles (PALS) program aimed at prevention of alcohol-, tobacco-, and other drug (AOTD) abuse. Prevention of alcohol-, tobacco-, and other drugs is rather commonplace in secondary schools. For instance, in the US, students typically get into prevention programs more than once in their school career (Kumar et al., 2013) and European schools are also encouraged to take action in promoting students' health (World Health Organiasation, 2011). Teachers attended a 1-day workshop about adaptive teaching by means of: modifying time, increasing or decreasing the number of items to be learned or completed, increasing the level of support, changing the input or the way the material is presented, changing the output, adapting the amount of active participation, changing to alternate goals and expectations, adapting the level of difficulty for each individual, and providing different instruction and materials. In addition, teachers learned about alternative learning styles and disabilities. PALS materials were developed by the research team to match students' specific needs and related abilities. In a quasi-experimental study, four grade 6–8 teachers taught the 10 PALS intervention lessons to their classes and PALS team members taught another 24 classes. School officials suggested a convenient comparison group receiving the traditional prevention program. In reference to the control group, the PALS program had a large significant effect of *d* = +1.374 on students' knowledge of the effects of ATOD (see Table 2). These results were replicated in a second, within-group repeated measures design. Although the findings seem promising, more information is needed about how the approach was implemented; in the paper, it is unclear how teachers applied the information from the training in their instruction. Moreover, replication of the findings in a study in which teachers teach all project lessons may also help clarify whether the effects of the intervention were affected by the fact that project staff taught most lessons in the experimental condition.

We only selected two studies using a generic approach to differentiated instruction and the effects of the studies described above differ considerably regarding their intervention, school subject, and findings. This makes it hard to estimate the overall effectiveness of generic approaches. The study of Huber seems promising, but unfortunately, the study of Vogt and Rogalla did not lead to positive achievement effects for students across the primary and secondary school group. More studies are needed to gain insight in how teachers could effectively and efficiently be supported or coached to master the multifaceted approach of differentiated instruction.

#### Studies on Differentiated Instruction Using Homogeneous Clustering

A number of selected studies use a macro-adaptive approach to differentiated instruction (Richards and Omdal, 2007; Altintas and Özdemir, 2015a,b; Bal, 2016; Bikić et al., 2016). Of these studies, the study of Richards and Omdal (2007) has the most robust design. In this study, first year students were randomized over 14 classes and then classes were randomly assigned to conditions. Within the experimental condition, the science content for ability groups was adapted to students' learning needs by means of tiering. To study the effectiveness of the approach, 194 students were randomly assigned to classes in which the teachers used tiered content, while 194 other students were in the control group that worked with the midrange curriculum for 4 weeks. Each teacher was assigned at least one treatment and one control class. After a pretest, students in the experimental condition were assigned to three ability groups: a low background knowledge group (around the lowest scoring 10 percent of all students), a midrange group (about 80 percent), and a high background group (the highest scoring 10 percent). One of the researchers produced the instructional materials for the study. To develop the differentiated materials, first core instructional materials were developed that were aimed at the midrange group. Next, the content was differentiated for the low and high background students. Adaptations were made to the depth of content, the degree of teacher dependence and structuring, the number of steps, the skills, time on task, the product, and the available resources. Students were asked to work together within their tiers. There was an overall small significant effect of the intervention of *d* = +0.284 in favor of the tiering condition (see Table 2). Closer analyses of subgroup results (see Table 2) show that this is particularly due to a large effect for the low background learners of *d* = +1.057. For high-range learners, differences between the control condition and the experimental condition are near to zero (*d* = +0.077), although this may be partly due to a ceiling effect on the test. The authors conclude that curriculum differentiation through tiered assignments can be an effective way to address the needs of low achieving students. They recommend, however, that it should be accompanied by professional support and that teachers who design the tiers should have substantial subject matter knowledge and experience with learners with different needs.

In the study by Bikić et al. (2016), the effectiveness of differentiated instruction of geometry content within a problem-based learning approach is studied. In the quasi-experiment, the authors compare an approach in which students solved mathematics problems on three levels differing in complexity using problem-based learning to a control condition. The study design is not described in detail, but since the authors state “students of the experimental group and control group were not the students from the same school” it seems that schools were allocated to an experimental or control condition to study the effectiveness of the approach. Within the experimental condition, 88 secondary school students were assigned to three groups (low- average-, or high-achievers) based on an initial test, and then worked on adapted levels of geometry problems for 16 lessons before completing a final test. An example of the differentiated materials in the paper shows that the three ability groups all received a different task (which was a variation of the same task differing in complexity). Unfortunately, it is not described how the students exactly processed the content. In the control condition, 77 other students were taught in the usual, traditional manner. Students in the ability grouping condition outperformed the control students with a moderate positive effect of *d* = +0.539 (see Table 2). Subgroup analyses indicate that the approach was most effective for average ability students; students in the high achieving group did not outperform high achieving students in the control group. Do note however that the high achieving groups were small (12 exp. vs. 14 contr. students), hence, these results should be interpreted with caution. More research would be needed to clarify to which extent the differentiated content improved the effectiveness of the problem-based learning approach.

A different grouping approach is one based on preferred learning styles. In the study of Bal (2016), grade 6 students completed an algebra pre-test as well as filling out a learning style inventory (kinesthetic, visual, affective learning styles). Algebra-learning materials an activities are adapted for two tiers; for low performing students and high performing students, also adapted for different learning styles of students in the experimental group. Despite the fact that there are reasons not to use learning styles as a distinction between students (see e.g., Kirschner et al., 2018), the authors did find large positive effects of the tiering approach after 4 weeks of teaching (*d* = + 1.085, see Table 2). Do note however that ANCOVA results were used to calculate the effects which may lead to some positive bias in this estimate. Based on information from student-interviews presented in the paper, it seems that students experienced success in learning and enjoyed the materials and activities developed for the experimental condition. It is unclear however, how the materials and activities were made more appropriate for students' readiness (and learning style) and how they differed from the approach in the control condition that used traditional teaching. In that sense, it is difficult to judge what caused these positive findings. In another study on mathematics by Altintas and Özdemir (2015a,b), teachers assessed students' preferred learning modalities by taking a multiple intelligences inventory. The data obtained from the inventory were used to determine the students' project topics, to select the teachers' teaching strategies, and to determine the relevant factors for motivating students. The effectiveness of the approach, which was originally designed for gifted students, was evaluated in a sample of 5 to 7th grade students in Turkey. After pretesting, one class of students was allocated to the experimental condition and one class of the same grade formed the control group. The authors report a very large effect of the intervention after six practices lasting 7 weeks each when compared to classes working with the Purdue model for both grade 6 and grade 7 students (*d* = +4.504 across subgroups, see Table 2). However, it is difficult to discern what exactly caused this finding. Little information was provided about how exactly the teachers planned and executed the lessons and how students' activities and objectives were matched to their dominant intelligences, nor was there much information about possible confounding factors. In addition, since the researcher who developed the multiple intelligences theory admits that the theory is no longer up to date (Gardner, 2016), one could question whether learning preferences could be better determined based on another distinction.

In summary, from the studies we found on the effectiveness approaches to differentiated instruction using homogeneous clustering, we could infer that overall small to medium sized effects (and in some cases also large effects) of the approach on student achievement can be achieved in beta subjects. The study of Altintas and Özdemir shows a very large effect of this approach and the study of Bal also shows large effects. However, before we can corroborate these findings, more information would be needed. When we look at the operationalizations of differentiated instruction in the two larger studies, we see that teachers used variations of learning tasks that were designed to better match the learning needs of different ability groups. Differential effects for student outcomes are somewhat variable; the results are most profound for the low achieving group in the study by Richards and Omdal (2007), and for the low and average achieving group in the study of Bikić et al. (2016). In both studies, effectiveness for the high achieving group seemed negligible.

#### Studies on Mastery Learning

In two included studies, mastery learning was used to boost student achievement in physics and mathematics. The quasi-experimental studies reporting on mastery learning approaches in secondary education used randomization of schools to conditions and were both performed in African schools (Wambugu and Changeiywo, 2008; Mitee and Obaitan, 2015). In the papers, the authors describe similar characteristics of mastery learning in their theoretical framework, such as specifying learning goals, breaking down the curriculum into small units, formative assessment, using corrective instruction for students who did not reach mastery, and retesting. This process continues until virtually all the students master the taught material (Mitee and Obaitan, 2015), which emphasizes its aim of convergent differentiation. Mittee and Obaitan report a large effect of the mastery learning approach of *d* = +1.461 based on an experiment in which about 400 students from four schools were allocated to a mastery learning or a control condition (see Table 2). Wambugu and Changeiywo randomly divided four classes from four schools over the mastery learning or the experimental condition. Comparing the results on the physics achievement test of the two experimental classes a two control classes, they found a large effect of mastery learning (*d* = +1.322 based on the findings of an ANOVA, see Table 2). However, do note that pretests were only available for two out of four classes (one control and one experimental).

Unfortunately, the information on the mastery learning approach in the lessons is rather limited in both papers. Therefore, it is difficult to judge how such large achievement gains can be reached by implementing mastery learning in secondary education. Nevertheless, we can extract a number of recommendations: First, both studies use corrective instruction for helping students gain mastery. Secondly, in both studies the authors refer to some type of collaborative learning in the corrective instruction phase. Lastly, Wambugu and Changeiywo note that the time needed to develop the learning objectives, formative tests, and corrective activities is considerable so teachers may want to work together in teacher teams to achieve these goals. More high-quality research is needed to replicate these findings and to gain insight in how teachers can apply this approach in practice.

#### Studies on Individualized Differentiated Instruction

The large-scale quasi-experimental study on differentiated reading instruction in middle schools by Little et al. (2014) used individualized adaptations to address students' learning needs. They used a program called the Schoolwide Enrichment Model-Reading Framework (SEM-R) to support students' reading adaptively. The SEM-R approach consists of three phases: (1) short read-alouds by the teacher (“Book Hooks”) and brief discussions about books, (2) students read independently in self-selected, challenging books while the teacher organizes individualized 5- to 7-min conferences with each student once every 1 to 2 weeks, (3) interest-based and more project-oriented activities. Professional development of teachers included workshops as well as classroom support from project staff. The focus of the intervention was on phases 1 and 2. Teachers were expected to implement SEM-R on a daily basis for about 40 to 45 min per day or 3 h per week. In a cluster-randomized design executed in four middle schools with 2,150 students, the effectiveness of the approach was compared to that of traditional teaching. The effects of the approach varied considerably across the different schools. The authors reported that, for the reading fluency outcome, SEM-R students significantly outperformed their control counterparts in two out of four schools. The standardized mean differences ranged from about −0.1 to +0.3 between the schools (see Table 2). The authors conclude that the intervention was at least as effective as traditional instruction. However, the wide range of implementations and effects on student outcomes between classes and schools illustrates the difficulty of implementing intensive forms of individualization in practice.

In the survey study of Smit and Humpert (2012), the authors assessed which teaching practices teachers used to differentiate their teaching. In this sub-study of the project “Schools in Alpine Regions,” teachers from 8 primary schools and 14 secondary schools in the rural Alpine region of Switzerland participated. Teachers responded to a teacher questionnaire about differentiated instruction. They mainly reported to make adaptations at the individual level by, for instance, providing students with individual tasks (tiered assignments), adapting the number of tasks, or providing more time to work on tasks. Teachers often used “learning plans” as well as tasks in which students could take individual learning trajectories varying the content or learning rate. Flexible grouping was less common and alternative assessments were very rare. Peer tutoring occurred frequently, and tiered assignments were very common. On average, 38% of teachers' weekly lessons were differentiated. The authors conclude that teachers in their sample, on average, did not execute very elaborate differentiated instruction. Moreover, no significant relation between differentiated instruction and student achievement was found for neither a standardized language test (*d* = −0.092) nor a standardized mathematics test (*d* = −0.085, see Table 2). Following the survey study, an intervention study was executed with 10 of the schools that were included in the survey-study. In this study (that was not included in our selection since it was not published in an academic journal), teachers participated in workshops and team meetings and logged their learning experiences in portfolios. Teachers barely progressed in their differentiated instruction during the 2.5-year project (Smit et al., 2011). Nevertheless, a high pedagogical team culture in schools was found to have a positive influence teachers' differentiated instruction (Smit et al., 2011; Smit and Humpert, 2012), and as such may be one of the keys to achieve improvement.

Overall, it seems that it is rather difficult to boost the achievement of the whole class by means of individualized approaches. However, as Little et al. (2014) suggest, individualization may be used as an approach to increase students' engagement with the learning content. A drawback of the approach may be that the requirements for organizing and monitoring learning activities by the teacher in individualized approaches could leave less time for high quality pedagogical interaction. Possibly, future research on individualization supported by digital technology may open up more possibilities for this approach to have high impact on student achievement (Education Endowment Foundation, n.d.).

#### Studies on Differentiated Instruction Using Heterogeneous Clustering

One of the included studies used differentiated instruction within mixed-ability learning settings. In the study by Mastropieri et al. (2006), grade eight students worked on science assignments in groups of two or three. Peer-mediated differentiated instruction and tiering was used to adapt the content to students' learning needs within the groups. The authors developed three tiers of each assignment varying in complexity. Within the peer groups, students could work on activities on their own appropriate level and continue to the next level once proficiency was obtained. All lower ability level students—including students with learning disabilities—were required to begin with the lowest tier. In the experiment, 13 classes with a total of 216 students were assigned to the peer-mediated differentiated content condition or a teacher-led control condition. The researchers divided the classes in such a way that each teacher taught at least one experimental and one control classroom. After about 12 weeks, a small positive effect was found in favor of the peer-mediated condition with tiered content on both the unit test and the high stakes end of year test (respectively *d* = + 0.466 and *d* = + 0.306, see Table 2). The overall effect of *d* = +0.386 is comparable to that of the tiering intervention of Richards and Omdal (2007) discussed earlier. The effect is slightly higher, but this may also partly be affected by the use of adjusted means. In any case, more research is needed to disentangle the effects of the peer-learning and the differentiated content.

#### Studies on Differentiated Instruction in Flipped Classrooms

In flipped classroom instruction, content dissemination (lecture) is moved outside of the classroom, typically by letting students watch instructional videos before the lesson. This opens up more time for active learning inside the classroom (Leo and Puzio, 2016). This format implies differentiation of learning time and pace *before the lesson* since students may rewind, pause or watch the video's multiple times according to their learning needs. However, whether the activities *during the lesson* encompass our operationalization of differentiated instruction (see Table 1) varies. From a recent meta-analysis on flipping the classroom (Akçayir and Akçayir, 2018), we found one study in secondary education in which remediation in the classroom was mentioned as being part of the intervention. Bhagat et al. (2016) report on a quasi-experiment in which 41 high school students were assigned to a classroom using flipping-the-classroom and 41 students were in the control condition. The experimental group underwent “flipped” lessons on trigonometry for 6 weeks, while the control group followed similar lessons using the conventional learning method. Students in the flipped condition watched videos of 15–20 min before the lesson. During the lesson, students discussed problems collaboratively and, in the meantime, students who needed remediation were provided with extra instruction. After the intervention, students from the flipped classrooms outperformed their counterparts on a mathematics test and were more motivated. The authors report a large effect of the intervention on students' mathematics achievement based on analysis of covariance. However, the combined effect across the subgroup mean differences is modest *d* = 0.376, see Table 2). On average, experimental students of all abilities performed better, except for high achievers who did not significantly outperform the control group. These differential effects should be interpreted with caution because of the limited number of students in the subgroups. The pro of this study is that it gives some insights in the benefits of differentiated instruction embedded in an innovative approach to teaching. Yet, the authors did not specify clearly what the remediation and collaborative learning in the classroom consisted of and cannot disentangle effects of different elements of the intervention. More research would be needed to clarify the role and effectiveness of differentiated instruction in flipped settings.

### Contextual and Personal Variables

As we discussed in the theoretical framework, many variables may influence teachers' implementation of differentiated instruction. We hoped to find evidence for this assumption in our selection of papers. However, in general, little information was provided about contextual and personal factors such as school, class, or teacher characteristics.

In our sample of studies, differentiated instruction was mostly applied to teaching mathematics and science. Additionally, there were also papers on literacy and social sciences. No clear differences in effectiveness could be observed between the subjects. Students varied in background characteristics across the studies. In the study by Little et al. (2014), for instance, about 48 to 77 percent of students were from low SES. In the study by Mastropieri et al. (2006), many ethnicities were represented. In the studies by Huber et al. (2009), students were mostly European-American. Student ages varied from about 11 to 17 years old (see Table 2). Teacher characteristics were rarely reported. In the study by Mastropieri et al. (2006), relatively inexperienced teachers participated with a mean of about 3 years in their current position, and in the studies by Vogt and Rogalla (2009) and Smit and Humpert (2012), years of teaching experience varied considerably, with an average of about 15 to 17 years.

The only variable that is rather consistent across the studies is that teachers in the included studies relied considerably on external sources of information or support to help them implement differentiated instruction within their classrooms. In most of the selected studies, the research team developed materials for students, and teachers were instructed or coached in implementing the interventions (see Table 2). Although we aimed to select practical interventions, little information is provided about whether teachers were able to successfully execute the differentiated instruction practices independently in the long run.

### Overall Effects of Differentiated Instruction

Ideally, combining our narrative reflection on the included papers with a meta-analysis of the findings would give us an answer as to how effective within-class differentiated instruction in secondary education may be. However, unfortunately, the number of papers that remained after applying our selection criteria is limited and the studies are heterogeneous in nature so meta-analyses of results should be interpreted with caution. To inform the readers however, we did add a forest plot with an overview of the average effect size of each individual study to the appendix (see Appendix D). In Table 2 the effects and intermediate calculations for individual studies are described. A summary effect across all studies is also reported (*d* = +0.741; 95% CI = 0.397–1.1085; *Q* = 507.701; *df* = 14; *p* < 0.01). The *p*-value of the *Q* statistic was significant which may indicate heterogeneity of the papers meaning that the true effects of the interventions may vary. Noticeably, the largest studies in our sample show small positive effects of differentiated instruction. In contrast, the relatively small studies reported on large effects, and the other studies mostly show moderate effects of the approach. A cumulative analysis (see Appendix D) illustrates that the small study by Altintas and Özdemir (2015a,b) considerably shifts the point estimate of the effect size in the positive direction. Excluding this outlier, the summary effect of differentiated instruction is *d* = +0.509 (95% CI = 0.215–0.803; see Appendix D). A funnel plot was made to check for publication bias (see Appendix E). Using Duval and Tweedie's Trim and Fill method (Duval and Tweedie, 2000), no adjusted values were estimated. This indicates that there is no evidence of publication bias. These analyses give some information about the range of effects that can be achieved with differentiated instruction interventions ranging. However, unquestionably, more information is needed before drawing a more definitive conclusion about the overall and relative effects of different approaches to differentiated instruction in secondary schools.

### Suggestions for Reporting on Differentiated Instruction Interventions

One of the issues we encountered when performing this review, was that interventions and research methodologies were often described rather briefly. In addition, relevant context information was frequently missing. This is problematic, not only from a scientific point of view, but also to judge the transferability of the findings to practice. Therefore, we encourage researchers to diligently report on the methods and analytical techniques they used and to be specific about the outcomes that led to their conclusions (see e.g., Hancock and Mueller, 2010). Except for this general suggestion, we would like to provide a number of specific recommendations for reporting on differentiated instruction interventions (see Appendix F).

## Conclusion and discussion

The most important conclusion from our systematic review of the literature is that there are too few high-quality studies on the effectiveness of differentiated instruction in secondary education. Only 12 studies from 14 papers were selected after applying strict selection criteria to a large amount of literature on the topic. As expected, we found papers on various operationalizations of differentiated instruction like homogeneous grouping, differentiated instruction in peer-learning, and individualization. However, even within the most well-known approaches like ability grouping, the empirical evidence was limited. High quality teacher-led differentiated instruction studies in secondary education are scarce, although the literature on ICT-applications for differentiated instruction seems to be on the rise. This paucity has not changed much after our search, although there are some recent interesting endeavors for teacher professionalization in differentiated instruction (Brink and Bartz, 2017; Schipper et al., 2017, 2018; Valiandes and Neophytou, 2018) and there have been some recent small-scale studies including aspects of differentiated instruction (Sezer, 2017; Adeniji et al., 2018). This paucity is remarkable given the large interest for the topic of differentiated instruction in both the literature as well as in policy and practice. Apparently, the premises of differentiated instruction seems substantial enough for schools and policy makers to move towards implementation before a solid research base has been established. On the one hand, this seems defendable; differentiated instruction matches the ambitions of educationists to be more student-oriented and to improve equity among students. In addition, there is prior research showing benefits of approaches like ability grouping and mastery learning for K-12 students' achievement (Guskey and Pigott, 1988; Kulik et al., 1990; Kulik, 1992; Lou et al., 1996; Hattie, 2009; Steenbergen-Hu et al., 2016). Furthermore, the ideas behind differentiated instruction are in line with approaches which have repeatedly been linked to better learning such as having students work on an appropriate level of moderate challenge according to their “zone of proximal development” and matching learning tasks to students' abilities and interests to create “flow” (Tomlinson et al., 2003). On the other hand, more research on different operationalizations of differentiated instruction is needed to help teachers and policy makers to determine which approaches are helpful for students of different characteristics and to gain insight in how these could be implemented successfully. From prior research in primary education, we know that it is likely that not all approaches have comparable effects, and that effects for low- average- and high ability students may vary (Deunk et al., 2018). Our current review shows that there is much work to be done in order to further clarify which approaches work and why within the context of secondary education.

Having said that, the studies that we did find do give us some directions about the expectations we may have about the effectiveness of differentiated instruction in secondary education. Most well-designed studies in our sample reported small to medium-sized positive effects of differentiated instruction on student achievement. This finding is comparable to the moderate effects found in most differentiated instruction reviews (e.g., Kulik, 1992; Lou et al., 1996; Steenbergen-Hu et al., 2016) and other studies on educational interventions (Sipe and Curlette, 1996). The overall effect in our study is a bit higher than in prior reviews, possibly due to the inclusion of various approaches to differentiated instruction, including mastery learning and more holistic approaches. Although we cannot give a conclusive answer about the effectiveness of differentiated instruction in secondary education, most of the included studies do illustrate the possibility of improving student achievement by means of differentiated instruction.

Moreover, the selected papers give insight in the many different ways that differentiated instruction can be operationalized and studied in secondary education. For instance, a number of studies used generic training of teachers in principles of differentiated instruction. Based on the findings, we would suggest that more research is needed to study how teachers can adequately be guided to implement such holistic approaches into their daily teaching (compare practicality theory by Janssen et al., 2015). Alternatively, in four of the selected studies homogeneous clustering by means of tiering and ability grouping was used as a structure for differentiated instruction. For the subgroups, learning content was adapted to better fit the needs of the students (Richards and Omdal, 2007; Altintas and Özdemir, 2015a,b; Bal, 2016; Bikić et al., 2016). Medium to large positive effects were reported of such an approach, indicating this may be one of the ways teachers may address differentiated instruction. This finding is comparable to findings on ability grouping in the meta-analyses by Steenbergen-Hu et al. (2016) and Lou et al. (1996). The effects were somewhat larger compared to those in the studies in primary education discussed by Deunk et al. (2018) and Slavin (1990a). One possible explanation might be that some of the studies mentioned in those previous reviews may have included grouping without any instructional adaptations, which was excluded from the current review. Also, in our selected papers on homogeneous clustering, researcher-developed outcome measures were used. Researcher-developed measures have previously been associated with larger effects than standardized measures (Slavin, 1987; Lou et al., 1996). Turning to another approach, two studies were reviewed on the effectiveness of mastery learning. The authors reported large effects of mastery learning on student achievement. However, since the research methods were not thoroughly described in the papers, we cannot say much about the quality of the intervention nor the implementation. Two other studies focused on individualization. Overall, small and non-significant effects of this approach were found. It could be that teachers grapple with the organizational requirements of individualized instruction (Education Endowment Foundation, n.d.). Additionally, a study was found that successfully embedded differentiated instruction in a peer-learning setting by means tiered content matching students' learning needs (Mastropieri et al., 2006). Lastly, one of the studies embedded remediation and collaboration in a flipped-classroom format illustrating how differentiated instruction can be applied within different approaches to teaching (Bhagat et al., 2016).

Unfortunately, in only three studies, authors reported on differential effects for subgroups of students within classes. This makes it difficult to judge which differentiated instruction approach is most suitable for whom. In the studies (Richards and Omdal, 2007; Bhagat et al., 2016; Bikić et al., 2016) that did report effects for subgroups, the interventions were shown to be most beneficial for low achieving (and in case of Bikić also the average achieving) subgroups of students, even though the learning content was adapted to better match the needs of other students too. However, it remains unclear whether this was caused by the differentiated instruction, by the fact that the teachers directed more attention toward low performing students, or by the fact that the outcome measures did not match the adapted content. In addition, the subgroups were relatively small, limiting the power of the findings. Therefore, more empirical evidence is needed about the implementation and relative effects of differentiated instruction to further inform the “differentiation-dilemma” of how to best divide time over students with different needs (Denessen, 2017).

Regarding the contextual and personal variables across studies, students' age, the school subjects and teaching experience of teachers varied. The fact that positive results have been replicated in several settings with different populations, gives a first indication that the approach may be transferable across different contexts (Petticrew and Roberts, 2006). One consistent finding across the studies is that teachers relied on external support to implement within-class differentiated instruction during the interventions. This is to be expected, since prior reviews found that implementing differentiated instruction is quite complex for teachers and that they may need considerable guidance to get it right (Tomlinson et al., 2003; Subban, 2006; Van Casteren et al., 2017). Previous studies show that teachers receiving more professional development in differentiated instruction perceive higher efficacy and adapt their teaching to students more often (Dixon et al., 2014; Suprayogi et al., 2017).

The contribution of the current review to existing knowledge of the effects of differentiated instruction on students' achievement in secondary education is as follows: First, it provides an overview of theoretical concepts and operationalizations of differentiated instruction in the classroom. Next, it shows that a systematic review of the literature leads to a limited body of evidence regarding the effectiveness of within-class differentiated instruction in secondary education. This overview of the state of the art within this theme may inform further research initiatives. Additionally, the study addresses some contextual and personal factors that may affect teachers' differentiated instruction.

### Limitations

The most salient drawback of the review is the limited number of studies that were included. On the one hand, it is unfortunate that the limited number of selected papers makes it difficult to come to definitive conclusions about the effectiveness of within-class differentiated instruction. On the other hand, the importance of using systematic reviews to identify research gaps to inform further development of the field should not be underestimated (Petticrew and Roberts, 2006). Defining consistent criteria for the selection of the best evidence available—as we have done in this study—may limit the number of selected studies but does help to ensure that the studies that are selected are highly informative (Slavin, 1995). The limited number of studies we found is just about comparable to the number of within-class approaches that were selected in a recent review of between-class and within-class differentiated instruction in primary education (Deunk et al., 2018). We only included studies in which student achievement was reported as an outcome measure. In future research, adding other types of outcomes and other types of study designs could add to the breadth of the research base.

Another limitation has to do with the quality of the selected papers and consequently with our approach to the analyses. First, the fact that we did not locate any truly randomized designs necessitates caution in interpreting the findings. Potential biases are likely to be greater for non-randomized studies compared to randomized trials (Higgins and Green, 2011). Second, the number of participants at the level of randomization (often the classroom level) was mostly low. Furthermore, it was sometimes difficult to determine the quality of the studies due to a lack of information in the papers. We tried to gain insight in the differentiated instruction interventions, but often essential information was omitted. Also, the conversion to Cohen's *d* could not always be done using an identical approach across the different studies. Must studies reported pre- and/or post-scores on achievement tests that we could use to calculate the effects in a rather straightforward manner, but in a few cases we had to estimate effects based on other types of information (for instance adjusted means or analyses of variance) which may complicate comparability across studies. Another drawback is that authors sometimes provided the outcomes of subgroups (for instance classes or ability groups within classes), sometimes only outcomes of the experimental conditions, or sometimes both. In the case of differentiated teaching, researchers should clearly explain their aims regarding which students they want to support (convergent or divergent). And if the aims differ per subgroup, they should ideally report these separate effects too. To inform future research on the topic, we have suggested some reporting guidelines that may help to clarify the content of future approaches to differentiated instruction and how they were studied in the Appendix.

A final limitation, inherent to a topic that is so multifaceted, is that the choices we have made in how we defined within-class differentiated instruction have influenced our selection of the literature and, thus, should be considered when interpreting the findings. The existing literature is marked by different ways of defining and operationalizing differentiated instruction (Suprayogi et al., 2017; Deunk et al., 2018). As such, our review may differ from the operationalizations of other authors. In addition, other ways to adapt teaching to students' learning needs are also certainly interesting to consider by teachers who want to better align teaching to students' needs. For example, the use of scaffolding techniques in which instruction is broken up in chunks, and instruction in each chunk is provided contingent to students' level of understanding is a promising instructional technique (Van de Pol et al., 2010, 2015). In addition, formative assessment is a helpful starting point for differentiated instruction or other types of adaptive teaching (Kingston and Nash, 2011). Furthermore, as discussed in the theoretical framework, differentiated instruction is a broad construct that adds up as a sum of its parts including lesson planning, differentiated instruction, evaluation and general high-quality teaching behaviors. We could not include all these factors into the working definition used to select and synthesize the studies. Therefore, readers should keep in mind that in order to understand differentiated instruction comprehensively and apply it in practice, there is more to it than just executing a differentiated lesson. A thoughtful approach using different steps starting from planning to evaluation including high quality teaching behaviors is key.

### Recommendations for Research and Practice

We would like to urge researchers to further study the impact and implementation of differentiated instruction. First, reviews and meta-analyses combining quantitative and qualitative information on the effects of different approaches to differentiated instruction for different outcomes may add further to the current knowledge base (Dixon-Woods et al., 2005). When more quantitative studies are located, this enables more statistical possibilities that can be used to gain insight in differential effects and predictive characteristics of different student outcomes (Lou et al., 1996; Moeyaert et al., 2016; Deunk et al., 2018). And qualitative studies may help us understand how teachers differentiate and how their subjective experiences in the classroom influence their differentiated instruction (Civitillo et al., 2016). In addition, authors may want to add studies on affective student outcomes as well. For example, students may have better attitudes and motivation in differentiated classes in which teaching better matches their learning needs (Kulik and Kulik, 1982; Lou et al., 1996; Maulana et al., 2017; Van Casteren et al., 2017).

Second, future studies on the development and evaluation of differentiated instruction interventions could add to the knowledge base about how to reach differentiated instruction's potential in practice. In order to support teachers, specific coaching on the job by experienced peers or external coaches or other types of professionalization may help to develop awareness and implementation of differentiated instruction (Latz et al., 2009; Smit and Humpert, 2012; Parsons et al., 2018; Valiandes and Neophytou, 2018). Teachers should learn to reflect upon the decisions they make when adapting their teaching (Parsons et al., 2018). Moreover, teachers need team support and sufficient time to develop their differentiated instruction (Stollman, 2018). Research shows that teachers themselves are quite enthusiastic about bottom-up professionalization approaches like peer-coaching or professional learning communities (Van Casteren et al., 2017). Whatever approach one chooses, there are some characteristics which may facilitate the effectiveness of professionalization including: a focus on both content and pedagogical knowledge, sufficient duration of the intervention, initial training and follow-up sessions, a facilitation of collaboration and communication with colleagues and experts, constant on-site support and help during the implementation- and the development of personal skills for reflection and self-evaluation of teachers (Valiandes and Neophytou, 2018). In addition, teacher educators should be mindful of teacher differences themselves too by providing differentiated professionalization (Stollman, 2018). In this review, we did not include studies on the effectiveness of adaptive ICT applications on students' progress. However, ICT can play a significant role in the creation of student-centered learning environments when used as more than a simple add-on to regular teaching (Smeets and Mooij, 2001; Deunk et al., 2018). Some recent studies on adaptive or personalized ICT programs, digital pen technologies, and blended learning show that such interventions can support differentiated instruction and have positive effects on student achievement (Walkington, 2013; Chen et al., 2016; Van Halem et al., 2017; Ghysels and Haelermans, 2018), although more research is needed to assess for whom and for which type of outcomes these approaches are beneficial (Van Klaveren et al., 2017). In the studies in this review, fixed outcome measures were used to assess students' learning. Possibly, adaptive testing will provide more room for assessing differentiated growth trajectories in future studies (Martin and Lazendic, 2018).

Lastly, when aiming to gain further insight in the effectiveness of differentiated instruction, authors may want to reflect on how differentiated instruction is operationalized and measured. In prior research, teacher questionnaires were often used to assess teachers' differentiated instruction practices (Roy et al., 2013; Prast et al., 2015). In addition, classroom observations of differentiated instruction or adaptive teaching behavior have been used (Cassady et al., 2004; Van Tassel-Baska et al., 2006; Van de Grift, 2007). Alternatively, in our selection of papers, we found some interesting ways to determine how teachers differentiate. For example, using vignette or video tests (Vogt and Rogalla, 2009; Bruhwiler and Blatchford, 2011) or by means of teacher logs or observations (Little et al., 2014). Enriching measures of teacher behavior with information about the match of the behavior with students' needs may be another step forward (Van Geel et al., 2019). We would like to recommend authors to further develop, evaluate and apply measures for differentiated instruction that can be used to gain insight in how differentiated instruction is linked to various student outcomes.

## Data Availability Statement

The datasets generated for this study are available on request to the corresponding author.

## Author Contributions

AS-J set up the methods of the paper, analyzed the theoretical backgrounds and is responsible for the concept of the article, and together with co-authors, extracted data, performed the analyses, and wrote the paper. AM coordinated the selection of studies, worked on data selection and extraction, and contributed to writing the paper. MH-L and RM designed the overarching project, acquired funding for the execution, and contributed to the conceptualization of differentiated instruction and the review process.

### Conflict of Interest

The authors declare that the research was conducted in the absence of any commercial or financial relationships that could be construed as a potential conflict of interest.

## Footnotes

^1^We did not include search terms specifically referring to heterogeneous approaches in the search string. Although heterogeneous grouping may include differentiation, adaptiveness is often not the focus of these studies.

^2^Quasi-experimental studies in which experimental and control groups are well matched, and covariates that correlate strongly with pretests are used to adjust outcomes, can be a valuable source of information usable for meta-analyses (Slavin et al., 2008; Slavin and Smith, 2009), although the results of (especially small-scale) quasi-experimental studies should be evaluated with caution (Cheung and Slavin, 2016).

## References

[B1] Adeniji S. M. Ameen S. K. Dambatta B. U. Orilonise R. (2018). Effect of mastery learning approach on senior school students' academic performance and retention in circle geometry. Int. J. Instruct. 11, 951–962. 10.12973/iji.2018.11460a

[B2] Akçayir G. Akçayir M. (2018). The flipped classroom: a review of its advantages and challenges. Comput. Educ. 126, 334–345. 10.1016/j.compedu.2018.07.021

[B3] Altemueller L. Lindquist C. (2017). Flipped classroom instruction for inclusive learning. Br. J. Spec. Educ. 44, 341–358. 10.1111/1467-8578.12177

[B4] ^*^ Altintas E. Özdemir A. S. (2015a). The effect of the developed differentiation approach on the achievements of the students. Eurasian J. Educ. Res. 61, 199–216. 10.14689/ejer.2015.61.11

[B5] ^*^ Altintas E. Özdemir A. S. (2015b). Evaluating a newly developed differentiation approach in terms of student achievement and teachers' opinions. Educ. Sci. Theor. Pract. 15, 1103–1118. 10.12738/estp.2015.4.2540

[B6] ^*^ Bal A. P. (2016). The effect of the differentiated teaching approach in the algebraic learning field on students' academic achievements. Eurasian J. Educ. Res. 63, 185–204. 10.14689/ejer.2016.63.11

[B7] Best Evidence Encyclopedia (n.d.) Review Methods. Criteria for Inclusion in the Best Evidence Encyclopedia. Available online at: http://www.bestevidence.org/methods/criteria.htm

[B8] ^*^ Bhagat K. K. Chang C. Chang C. (2016). The impact of the flipped classroom on mathematics concept learning in high school. J. Educ. Technol. Soc. 19, 134–142. Available online at: https://psycnet.apa.org/record/2016-35586-003

[B9] ^*^ Bikić N. Maričić S. M. Pikula M. (2016). The effects of differentiation of content in problem-solving in learning geometry in secondary school. EURASIA J. Math. Sci. Technol. Educ. 12, 2783–2795. 10.12973/eurasia.2016.02304a

[B10] Blatchford P. Bassett P. Brown P. (2011). Examining the effect of class size on classroom engagement and teacher-pupil interaction: differences in relation to pupil prior attainment and primary vs. secondary schools. Learn. Instruct. 21, 715–730. 10.1016/j.learninstruc.2011.04.001

[B11] Borenstein M. Hedges L. V. Higgins J. P. T. Rothstein H. R. (2009). Introduction to Meta-Analysis. Chichester: John Wiley and Sons. 10.1002/9780470743386

[B12] Borenstein M. Hedges L. V. Higgins J. P. T. Rothstein H. R. (2010). A basic introduction to fixed-effect and random-effects models for meta-analysis. Res. Synth. Methods 1, 97–111. 10.1002/jrsm.12 26061376

[B13] Bosker R. J. (2005). De Grenzen van Gedifferentiëerd Onderwijs. Groningen: Rijksuniversiteit Groningen. Available online at: http://www.rug.nl/research/portal/files/14812458/bosker.pdf

[B14] Bray B. McClaskey K. (2013). Personalization vs. Differentiation vs. Individualization. (No. version 3). Available online at: http://www.personalizelearning.com/2012/04/explaining-chart.html

[B15] Brink M. Bartz D. E. (2017). Effective use of formative assessment by high school teachers. Pract. Assess. Res. Eval. 22, 1–10. Available online at: https://pareonline.net/getvn.asp?v=22&n=8

[B16] ^*^ Bruhwiler C. Blatchford P. (2011). Effects of class size and adaptive teaching competency on classroom processes and academic outcome. Learn. Instruct. 21, 95–108. 10.1016/j.learninstruc.2009.11.004

[B17] Cassady J. C. Neumeister K. L. S. Adams C. M. Cross T. L. Dixon F. A. Pierce R. L. (2004). The differentiated classroom observation scale. Roeper Rev. 26, 139–146. 10.1080/02783190409554259

[B18] Cavanagh S. (2014). What is personalised learning? Educators seek clarity. Education Week. Available online at: https://www.edweek.org/ew/articles/2014/10/22/09pl-overview.h34.html

[B19] Chen C. Tan C. Lo B. (2016). Facilitating English-language learners' oral reading fluency with digital pen technology. Interact. Learn. Environ. 24, 96–118. 10.1080/10494820.2013.817442

[B20] Cheung A. C. K. Slavin R. E. (2016). How methodological features affect effect sizes in education. Educ. Res. 45, 283–292. 10.3102/0013189X16656615

[B21] Cheung A. C. K. Slavin R. E. Kim E. Lake C. (2017). Effective secondary science programs: a best-evidence synthesis. J. Res. Sci. Teach. 54, 58–81. 10.1002/tea.21338

[B22] Civitillo S. Denessen E. Molenaar I. (2016). How to see the classroom through the eyes of a teacher: consistency between perceptions on diversity and differentiation practices. J. Res. Spec. Educ. Needs 16, 587–591. 10.1111/1471-3802.12190

[B23] Clarke D. Hollingsworth H. (2002). Elaborating a model of teacher professional growth. Teach. Teach. Educ. 18, 947–967. 10.1016/S0742-051X(02)00053-7

[B24] Cole R. Haimson J. Perez-Johnson I. May H. (2011). Variability in Pretest-Posttest Correlation Coefficients by Student Achievement Level. (NCEE Reference Report 2011-4033). Washington, DC: National Center for Education Evaluation and Regional Assistance, Institute of Education Sciences, U.S. Department of Education.

[B25] Corno L. (2008). On teaching adaptively. Educ. Psychol. 43, 161–173. 10.1080/00461520802178466

[B26] Coubergs C. Struyven K. Engels N. Cools W. De Martelaer K. (2013). Binnenklas-Differentiatie. Leerkansen Voor Alle Leerlingen. Leuven: Uitgeverij Acco.

[B27] De Jager T. (2013). Guidelines to assist the implementation of differentiated learning activities in south African secondary schools. Int. J. Inclus. Educ. 17, 80–94. 10.1080/13603116.2011.580465

[B28] De Neve D. Devos G. (2016). The role of environmental factors in beginning teachers' professional learning related to differentiated instruction. Sch. Effect. Sch. Improv. 27, 557–579. 10.1080/09243453.2015.1122637

[B29] Denessen E. J. P. G. (2017). Verantwoord Omgaan met Verschillen: Social-Culturele Achtergronden en Differentiatie in Het Onderwijs. [Soundly Dealing with Differences: Socialcultural Background and Differentiation in Education]. (Inaugural lecture). Leiden: Leiden University. Available online at: https://openaccess.leidenuniv.~nl/handle/1887/51574

[B30] Denessen E. J. P. G. Douglas A. S. (2015). Teacher expectations and within-classroom differentiation, in Routledge International Handbook of Social Psychology of the Classroom, eds Rubie-Davies C. M. Stephens J. M. Watson P. (London: Routledge; Taylor and Francis Group, 296–303.

[B31] Deunk M. I. Smale-Jacobse A. E. de Boer H. Doolaard S. Bosker R. J. (2018). Effective differentiation practices: a systematic review and meta-analysis of studies on the cognitive effects of differentiation practices in primary education. Educ. Res. Rev. 24, 31–54. 10.1016/j.edurev.2018.02.002

[B32] Dixon F. A. Yssel N. McConnell J. M. Hardin T. (2014). Differentiated instruction, professional development, and teacher efficacy. J. Educ. Gifted 37, 111–127. 10.1177/0162353214529042

[B33] Dixon-Woods M. Agarwal S. Jones D. Young B. Sutton A. (2005). Synthesising qualitative and quantitative evidence: a review of possible methods. J. Health Serv. Res. Policy 10, 45–53. 10.1177/135581960501000110 15667704

[B34] Duval S. Tweedie R. (2000). Trim and fill: a simple funnel-plot-based method of testing and adjusting for publication bias in meta-analysis. Biometrics 56, 455–463. 10.1111/j.0006-341X.2000.00455.x 10877304

[B35] Education Endowment Foundation (n.d.) Teaching Learning Toolkit An Accessible Summary of the International Evidence on Teaching 5-16 year-Olds. Available online at: https://educationendowmentfoundation.org.uk/evidence-summaries/teaching-learning-toolkit/

[B36] Edwards P. Clarke M. DiGuiseppi C. Pratap S. Roberts I. Wentz R. (2002). Identification of randomized controlled trials in systematic reviews: accuracy and reliability of screening records. Stat. Med. 21, 1635–1640. 10.1002/sim.1190 12111924

[B37] Gardner H. (2016). Multiple intelligences: prelude, theory, and aftermath, in Scientists Making a Difference, eds Sternberg R. J. Fiske S. T. Foss D. J. (New York, NY: Cambridge University Press). 10.1017/CBO9781316422250

[B38] Ghysels J. Haelermans C. (2018). New evidence on the effect of computerized individualized practice and instruction on language skills. J. Comput. Assist. Learn. 34, 440–449. 10.1111/jcal.12248

[B39] Guskey T. R. Pigott T. D. (1988). Research on group-based mastery learning programs: a meta-analysis. J. Educ. Res. 81, 197–216. 10.1080/00220671.1988.10885824

[B40] Hall E. F. (1992). Assessment for differentiation. Br. J. Spec. Educ. 19, 20–23. 10.1111/j.1467-8578.1992.tb00397.x

[B41] Hancock G. R. Mueller R. O. (2010). The Reviewer's Guide to Quantitative Methods in the Social Sciences. New York, NY: Routledge.

[B42] Hattie J. (2009). Visible Learning. A Synthesis of Over 800 Meta-Analyses Relating to Achievement. Oxon: Routledge.

[B43] Hertberg-Davis H. Brighton C. M. (2006). Support and sabotage: principals' influence on middle school teachers' responses to differentiation. J. Secondary Gifted Educ. 17, 90–102. 10.4219/jsge-2006-685

[B44] Higgins J.P.T. Green S. (eds). (2011). Cochrane Handbook for Systematic Reviews of Interventions Version 5.1.0 [updated March 2011]. The Cochrane Collaboration. Available online at: www.handbook.cochrane.org.

[B45] ^*^ Huber M. J. Workman J. Ford J. A. Moore D. Mayer T. (2009). Evaluating the prevention through alternative learning styles program. J. Drug Educ. 39, 239–259. 10.2190/DE.39.3.b 20196330

[B46] Imants J. Van Veen K. (2010). Teacher learning as workplace learning, in International Encyclopedia of Education, 3rd Edn. eds Peterson P. Baker E. McGaw B. (Oxford: Elsevier), 569–574. 10.1016/B978-0-08-044894-7.00657-6

[B47] Janssen F. Westbroek H. Doyle W. (2015). Practicality studies: how to move from what works in principle to what works in practice. J. Learn. Sci. 24, 176–186. 10.1080/10508406.2014.954751

[B48] Keuning T. Van Geel M. Frèrejean J. Van Merriënboer J. Dolmans D. Visscher A. (2017). Differentiëren bij rekenen: Een cognitieve taakanalyse van het denken en handelen van basisschoolleerkrachten [Differentiating in mathematics: a cognitive task analysis of primary school teachers' reflections and practices]. Pedagog. Stud. 94, 160–181. Available online at: http://pedagogischestudien.nl/download?type=document&identifier=640319

[B49] Kiley D. (2011). Differentiated Instruction in the Secondary Classroom: Analysis of the Level of Implementation and Factors that Influence Practice (Partial FULFILLMENT of the Requirements for the Degree of Doctor of Education). Kalamazoo: Western Michigan University.

[B50] Kingston N. Nash B. (2011). Formative assessment: a meta-analysis and a call for research. Educ. Meas. Issues Pract. 30, 28–37. 10.1111/j.1745-3992.2011.00220.x

[B51] Kirschner P. A. Claessens L. Raaijmakers S. (2018). Op de Schouders van Reuzen. Inspirerende Inzichten uit de Cognitieve Psychologie voor Leerkrachten. [On the Shoulders of Giants. Inspiring Insights from Cognitive Psychology for Teachers]. Meppel: Drukkerij Ten Brink Uitgevers.

[B52] Kulik C. C. Kulik J. A. (1982). Effects of ability grouping on secondary school students: a meta-analysis of evaluation findings. Am. Educ. Res. J. 19, 415–428. 10.3102/00028312019003415

[B53] Kulik C. C. Kulik J. A. Bangert-Drowns R. L. (1990). Effectiveness of mastery learning programs: a meta-analysis. Rev. Educ. Res. 60, 265–299. 10.3102/00346543060002265

[B54] Kulik J. A. (1992). An Analysis of the Research on Ability Grouping: Historical and Contemporary Perspectives. Research-Based Decision Making Series. National Research Center on the Gifted and Talented. Available online at: http://search.ebscohost.com.proxy-ub.rug.nl/login.aspx?direct=trueanddb=ericandAN=ED350777andsite=ehost-liveandscope=site

[B55] Kulik J. A. Fletcher J. D. (2016). Effectiveness of intelligent tutoring systems: a meta-analytic review. Rev. Educ. Res. 86, 42–78. 10.3102/0034654315581420

[B56] Kumar R. O'malley P. M. Johnston L. D. Laetz V. B. (2013). Alcohol, tobacco, and other drug use prevention programs in U.S. schools: a descriptive summary. Prev. Sci. 14, 581–592. 10.1007/s11121-012-0340-z 23404662 PMC3706520

[B57] Kyriakides L. Creemers B. Charalambous E. (2018). Equity and Quality Dimensions in Educational Effectiveness. Dordrecht: Springer International Publishing 10.1007/978-3-319-72066-1

[B58] Latz A. O. Speirs Neumeister K. L. Adams C. M. Pierce R. L. (2009). Peer coaching to improve classroom differentiation: perspectives from project CLUE. Roeper Rev. 31, 27–39. 10.1080/02783190802527356

[B59] Leo J. Puzio K. (2016). Flipped instruction in a high school science classroom. J. Sci. Educ. Technol. 25, 775–781. 10.1007/s10956-016-9634-4

[B60] ^*^ Little C. A. McCoach D. B. Reis S. M. (2014). Effects of differentiated reading instruction on student achievement in middle school. J. Adv. Acad. 25, 384–402. 10.1177/1932202X14549250

[B61] Lou Y. Abrami P. C. Spence J. C. Poulsen C. Chambers B. d'Apollonia S. (1996). Within-class grouping: a meta-analysis. Rev. Educ. Res. 66, 423–458. 10.3102/00346543066004423

[B62] Lyons L. C. (2003). Meta-Analysis: Methods of Accumulating Results Across Research Domains. Available online at: http://www.lyonsmorris.com/lyons/metaAnalysis/index.cfm

[B63] Ma W. Adesope O. O. Nesbit J. C. Liu Q. (2014). Intelligent tutoring systems and learning outcomes: a meta-analysis. J. Educ. Psychol. 106, 901–918. 10.1037/a0037123

[B64] Martin A. J. Lazendic G. (2018). Computer-adaptive testing: implications for students' achievement, motivation, engagement, and subjective test experience. J. Educ. Psychol. 110, 27–45. 10.1037/edu0000205

[B65] ^*^ Mastropieri M. A. Scruggs T. E. Norland J. J. Berkeley S. McDuffie K. Tornquist E. H. (2006). Differentiated curriculum enhancement in inclusive middle school science: effects on classroom and high-stakes tests. J. Spec. Educ. 40, 130–137. 10.1177/00224669060400030101

[B66] Maulana R. Helms-Lorenz M. Van de Grift W. J. C. M. (2015). Development and evaluation of a questionnaire measuring pre-service teachers' teaching behaviour: a rasch modelling approach. Sch. Effect. Sch. Improv. 26, 169–194. 10.1080/09243453.2014.939198

[B67] Maulana R. Helms-Lorenz M. Van de Grift W. J. C. M. (2017). Validating a model of effective teaching behaviour of pre-service teachers. Teach. Teach. Theor. Pract. 23, 471–493. 10.1080/13540602.2016.1211102

[B68] McQuarrie L. McRae P. Stack-Cutler H. (2008). Differentiated Instruction Provincial Research Review. Edmonton, AB: Alberta Initiative for School Improvement.

[B69] Mevarech Z. R. Kramarski B. (1997). IMPROVE: a multidimensional method for teaching mathematics in heterogeneous classrooms. Am. Educ. Res. J. 34, 365–394. 10.3102/00028312034002365

[B70] Mills M. Monk S. Keddie A. Renshaw P. Christie P. Geelan D. (2014). Differentiated learning: from policy to classroom. Oxford Rev. Educ. 40, 331–348. 10.1080/03054985.2014.911725

[B71] ^*^ Mitee T. L. Obaitan G. N. (2015). Effect of mastery learning on senior secondary school students' cognitive learning outcome in quantitative chemistry. J. Educ. Pract. 6, 34–38. Available online at: https://files.eric.ed.gov/fulltext/EJ1083639.pdf

[B72] Moeyaert M. Ugille M. Beretvas N. Ferron J. Bunuan R. Van den Noortgate W. (2016). Methods for dealing with multiple outcomes in meta-analysis: a comparison between averaging effect sizes, robust variance estimation and multilevel meta-analysis. Int. J. Soc. Res. Methodol. 20, 559–572. 10.1080/13645579.2016.1252189

[B73] Nokes-Malach T. Richey J. Gadgil S. (2015). When is it better to learn together? Insights from research on collaborative learning. Educ. Psychol. Rev. 27, 645–656. 10.1007/s10648-015-9312-8

[B74] Oakes J. (2008). Keeping track: structuring equality and inequality in an era of accountability. Teach. College Rec. 110, 700–712. Available online at: https://www.tcrecord.org/Content.asp?ContentId=14610

[B75] OECD (2012). Equity and Quality in Education. Supporting Disadvantaged Students and Schools. Paris: OECD Publishing 10.1787/9789264130852-en

[B76] OECD (2018). The Resilience of Students with an Immigrant Background. Factors that Shape Well-being. Paris: OECD Publishing 10.1787/9789264292093-en

[B77] Parsons S. A. Dodman S. L. Cohen Burrowbridge S. (2013). Broadening the view of differentiated instruction differentiation shouldn't end with planning but should continue as teachers adapt their instruction during lessons. Kappan 95, 38–42. 10.1177/003172171309500107

[B78] Parsons S. A. Vaughn M. Scales R. Q. Gallagher M. A. Parsons A. W. Davis S. G. (2018). Teachers' instructional adaptations: a research synthesis. Rev. Educ. Res. 88, 205–242. 10.3102/0034654317743198

[B79] Petticrew M. Roberts H. (2006). Systematic Reviews in the Social Sciences. A Practical Guide. Malden, MA: USA Blackwell publishing 10.1002/9780470754887

[B80] Pierce R. Adams C. (2005). Using tiered lessons in mathematics. Math. Teach. Middle Sch. 11, 144–149.

[B81] Prast E. J. Van de Weijer-Bergsma E. Kroesbergen E. H. Van Luit Johannes E. H. (2015). Readiness-based differentiation in primary school mathematics: expert recommendations and teacher self-assessment. Frontline Learn. Res. 3, 90–116. 10.14786/flr.v3i2.163

[B82] ^*^ Richards M. R. E. Omdal S. N. (2007). Effects of tiered instruction on academic achievement in a secondary science course. J. Adv. Acad. 18, 424–453. 10.4219/jaa-2007-499

[B83] Rock M. L. Gregg M. Ellis E. Gable R. A. (2008). REACH: a framework for differentiating classroom instruction. Prev. Sch. Fail. 52, 31–47. 10.3200/PSFL.52.2.31-47

[B84] Roy A. Guay F. Valois P. (2013). Teaching to address diverse learning needs: development and validation of a differentiated instruction scale. Int. J. Inclus. Educ. 17, 1186–1204. 10.1080/13603116.2012.743604

[B85] Scheerens J. (2016). Meta-analyses of school and instructional effectiveness, in Educational Effectiveness and Ineffectiveness, ed Scheerens J. (Dordrecht: Springer Science + Business Media), 175–223. 10.1007/978-94-017-7459-8_8

[B86] Schipper T. Goei S. L. de Vries S. van Veen K. (2017). Professional growth in adaptive teaching competence as a result of lesson study. Teach. Teach. Educ. 68, 289–303. 10.1016/j.tate.2017.09.015

[B87] Schipper T. Goei S. L. de Vries S. van Veen K. (2018). Developing teachers' self-efficacy and adaptive teaching behaviour through lesson study. International Journal of Educational Research, 88, 109–120. 10.1016/j.ijer.2018.01.011

[B88] Schleicher A. (2016). Teaching Excellence Through Professional Learning and Policy Reform: Lessons from Around the World. Paris: International Summit on the Teaching Profession; OECD Publishing 10.1787/9789264252059-en

[B89] Schofield J. W. (2010). International evidence on ability grouping with curriculum differentiation and the achievement gap in secondary schools. Teach. College Rec. 112, 1492–1528. Available online at: https://www.tcrecord.org/Content.asp?ContentId=15684

[B90] Schütz G. Ursprung H. Wößmann L. (2008). Education Policy and Equality of Opportunity, Vol. 61 (Kyklos: Wiley Blackwell), 279–308.

[B91] Sezer B. (2017). The effectiveness of a technology-enhanced flipped science classroom. J. Educ. Comput. Res. 55, 471–494. 10.1177/0735633116671325

[B92] Shute V. J. Rahimi S. (2017). Review of computer-based assessment for learning in elementary and secondary education. J. Comput. Assist. Learn. 33, 1–19. 10.1111/jcal.12172

[B93] Sipe T. A. Curlette W. L. (1996). A meta-synthesis of factors related to educational achievement: a methodological approach to summarizing and synthesizing meta-analyses. Int. J. Educ. Res. 25, 83–698. 10.1016/S0883-0355(96)80001-2

[B94] Slavin R. Smith D. (2009). The relationship between sample sizes and effect sizes in systematic reviews in education. Educ. Eval. Policy Anal. 31, 500–506. 10.3102/0162373709352369

[B95] Slavin R. E. (1986). Best-evidence synthesis: an alternative to meta-analytic and traditional reviews. Educ. Res. 15, 5–11. 10.3102/0013189X015009005

[B96] Slavin R. E. (1987). Mastery learning reconsidered. Rev. Educ. Res. 57, 175–214. 10.3102/00346543057002175

[B97] Slavin R. E. (1990a). Achievement effects of ability grouping in secondary schools: a best-evidence synthesis. Rev. Educ. Res. 60, 471–499. 10.3102/00346543060003471

[B98] Slavin R. E. (1990b). Mastery learning re-reconsidered. Rev. Educ. Res. 60, 300–302. 10.3102/00346543060002300

[B99] Slavin R. E. (1995). Best evidence synthesis. An intelligent alternative to meta-analysis. J. Clin. Epidemiol. 48, 9–18. 10.1016/0895-4356(94)00097-A 7853053

[B100] Slavin R. E. (2013). Effective programmes in reading and mathematics: lessons from the Best Evidence Encyclopaedia. Sch. Effect. Sch. Improv. 24, 383–391. 10.1080/09243453.2013.797913

[B101] Slavin R. E. Cheung A. (2005). A synthesis of research on language of reading instruction for English language learners. Rev. Educ. Res. 75, 247–284. 10.3102/00346543075002247

[B102] Slavin R. E. Cheung A. Groff C. Lake C. (2008). Effective reading programs for middle and high schools: a best-evidence synthesis. Read. Res. Q. 43, 290–322. 10.1598/RRQ.43.3.4

[B103] Slavin R. E. Lake C. Groff C. (2009). Effective programs in middle and high school mathematics: a best-evidence synthesis. Rev. Educ. Res. 79, 839–911. 10.3102/0034654308330968

[B104] Slavin R. E. Madden N. A. (2011). Measures inherent to treatments in program effectiveness reviews. J. Res. Educ. Effect. 4, 370–380. 10.1080/19345747.2011.558986

[B105] Smeets E. Mooij T. (2001). Pupil-centred learning, ICT, and teacher behaviour: observations in educational practice. Br. J. Educ. Technol. 32, 403 10.1111/1467-8535.00210

[B106] Smets W. Struyven K. (2018). Realist review of literature on catering for different instructional needs with preteaching and extended instruction. Educ. Sci. 8, 113 10.3390/educsci8030113

[B107] ^*^ Smit R. Humpert W. (2012). Differentiated instruction in small schools. Teach. Teach. Educ. 28, 1152–1162. 10.1016/j.tate.2012.07.003

[B108] Smit R. Humpert W. Obertüfer-Gahler R. Engeli E. Breuer-Brodmüller M. (2011). Differenzierung als Chance für kleine schulen - empirische befunde im längsschnitt, in Schule im Alpinen Raum, eds Müller R. Keller A. Kerle U. Raggl A. Steiner E. (Innsbruck: Studienverlag), 435–488.

[B109] Steenbergen-Hu S. Makel M. C. Olszewski-Kubilius P. (2016). What one hundred years of research says about the effects of ability grouping and acceleration on K−12 students' academic achievement: findings of two second-order meta-analyses. Rev. Educ. Res. 86, 849–899. 10.3102/0034654316675417

[B110] Stollman S. H. M. (2018). Differentiated Instruction in Practice: A Teacher Perspective. Leiden: ICLON, Leiden University Graduate School of Teaching.

[B111] Subban P. (2006). Differentiated instruction: a research basis. Int. Educ. J. 7, 935–947. Available online at: http://ehlt.flinders.edu.au/education/iej/articles/v7n7/Subban/BEGIN.HTM

[B112] Suprayogi M. N. Valcke M. Godwin R. (2017). Teachers and their implementation of differentiated instruction in the classroom. Teach. Teach. Educ. 67, 291–301. 10.1016/j.tate.2017.06.020

[B113] Swanson H. L. Lussier C. M. (2001). A selective synthesis of the experimental literature on dynamic assessment. Rev. Educ. Res. 71, 321–363. 10.3102/00346543071002321

[B114] Tieso C. L. (2003). Ability grouping is not just tracking anymore. Roeper Rev. 26, 29–36. 10.1080/02783190309554236

[B115] Tomlinson C. (2015). Teaching for excellence in academically diverse classrooms. Society 52, 203–209. 10.1007/s12115-015-9888-0

[B116] Tomlinson C. A. (1995). Deciding to differentiate instruction in middle school: one school's journey. Gifted Child Q. 39, 77–87. 10.1177/001698629503900204

[B117] Tomlinson C. A. (1999). Mapping a route toward differentiated instruction. Pers. Learn. 57, 12–16.

[B118] Tomlinson C. A. (2014). The Differentiated Classroom. Responding to the Needs of All Learrners, 2nd Edn. Alexandria, VA: ASCD.

[B119] Tomlinson C. A. Brighton C. Hertberg H. Callahan C. M. Moon T. R. Brimijoin K. (2003). Differentiating instruction in response to student readiness, interest, and learning profile in academically diverse classrooms: a review of literature. J. Educ. Gifted 27, 119–145. 10.1177/016235320302700203

[B120] Unesco (2017). A Guide for Ensuring Inclusion and Equity in Education. Paris: United Nations Educational, Scientific and Cultural Organization. Available online at: https://unesdoc.unesco.org/ark:/48223/pf0000248254

[B121] Valiande S. Koutselini M. I. (2009). (2009). Application and evaluation of differentiation instruction in mixed ability classrooms, Paper presented at the 4th Hellenic Observatory PhD Symposium (London: LSE, 25–26.

[B122] Valiandes S. Neophytou L. (2018). Teachers' professional development for differentiated instruction in mixed-ability classrooms: investigating the impact of a development program on teachers' professional learning and on students' achievement. Teach. Dev. 22, 123–138. 10.1080/13664530.2017.1338196

[B123] Van Casteren W. Bendig-Jacobs J. Wartenbergh-Cras F. Van Essen M. Kurver B. (2017). Differentiëren en Differentiatievaardigheden in Het Voortgezet Onderwijs. Nijmegen: ResearchNed.

[B124] Van de Grift W. J. C. M. (2007). Quality of teaching in four European countries: A review of the literature and application of an assessment instrument. Educ. Res. 49, 127–152. 10.1080/00131880701369651

[B125] Van de Grift W. J. C. M. Helms-Lorenz M. Maulana R. (2014). Teaching skills of student teachers: calibration of an evaluation instrument and its value in predicting student academic engagement. Stud. Educ. Eval. 43, 150–159. 10.1016/j.stueduc.2014.09.003

[B126] Van de Pol J. Volman M. Beishuizen J. (2010). Scaffolding in Teacher–Student interaction: a decade of research. Educ. Psychol. Rev. 22, 271–296. 10.1007/s10648-010-9127-6

[B127] Van de Pol J. Volman M. Oort F. Beishuizen J. (2015). The effects of scaffolding in the classroom: support contingency and student independent working time in relation to student achievement, task effort and appreciation of support. Instruct. Sci. 43, 615–641. 10.1007/s11251-015-9351-z

[B128] Van der Kleij F. Feskens R. C. W. Eggen T. J. H. M. (2015). Effects of feedback in a computer-based learning environment on students' learning outcomes. Rev. Educ. Res. 85, 475–511. 10.3102/0034654314564881

[B129] Van der Lans R. M. Van de Grift W. J. C. M. van Veen K. (2017). Individual differences in teacher development: an exploration of the applicability of a stage model to assess individual teachers. Learn. Individ. Diff. 58, 46–55. 10.1016/j.lindif.2017.07.007

[B130] Van der Lans R. M. Van de Grift W. J. C. M. van Veen K. (2018). Developing an instrument for teacher feedback: using the rasch model to explore teachers' development of effective teaching strategies and behaviors. J. Exp. Educ. 86, 247–264. 10.1080/00220973.2016.1268086

[B131] Van Geel M. Keuning T. Frèrejean J. Dolmans D. Van Merriënboer J. Visscher A. J. (2019). Capturing the complexity of differentiated instruction. Sch. Effect. Sch. Improv. 30, 51–67. 10.1080/09243453.2018.1539013

[B132] Van Halem N. Van Klaveren C. P. B. J. Cornelisz I. (2017). Oefent een leerling meer door niveaudifferentiatie? Het effect van data-gestuurde differentiatie op leerinspanning en de rol van eerder behaalde cijfers. [Does a learner practice more because of readiness-based differentiation? The effect of data-driven differentiation on learning effort and the role of prior grades]. Pedagog. Stud. 94, 182–195. Available online at: http://pedagogischestudien.nl/download?type=document&identifier=640298

[B133] Van Klaveren C. Vonk S. Cornelisz I. (2017). The effect of adaptive versus static practicing on student learning - evidence from a randomized field experiment. Econ. Educ. Rev. 58, 175–187. 10.1016/j.econedurev.2017.04.003

[B134] Van Tassel-Baska J. Quek C. Feng A. X. (2006). The development and use of a structured teacher observation scale to assess differentiated best practice. Roeper Rev. 29, 84–92. 10.1080/02783190709554391

[B135] ^*^ Vogt F. Rogalla M. (2009). Developing adaptive teaching competency through coaching. Teach. Teach. Educ. 25, 1051–1060. 10.1016/j.tate.2009.04.002

[B136] Walkington C. A. (2013). Using adaptive learning technologies to personalize instruction to student interests: the impact of relevant contexts on performance and learning outcomes. J. Educ. Psychol. 105, 932–945. 10.1037/a0031882

[B137] ^*^ Wambugu P. W. Changeiywo J. M. (2008). Effects of mastery learning approach on secondary school students' physics achievement. EURASIA J. Math. Sci. Technol. Educ. 4, 293–302. 10.12973/ejmste/75352

[B138] Wang M. C. Haertel G. D. Walberg H. J. (1990). What influences learning? A content analysis of review literature. J. Educ. Res. 84, 30–43. 10.1080/00220671.1990.10885988

[B139] Wilkinson S. D. Penney D. (2014). The effects of setting on classroom teaching and student learning in mainstream mathematics, English and science lessons: a critical review of the literature in England. Educ. Rev. 66, 411–427. 10.1080/00131911.2013.787971

[B140] World Health Organiasation (2011). European Action Plan to Reduce the Harmful Use of Alcohol 2012–2020. Copenhagen: World Health Organization Regional Office for Europe. Available online at: https://www.stap.nl/en/home/european-alcohol-policy.html

