# Supplementary material for: Differentiated Instruction in Secondary Education: A Systematic Review of Research Evidence
Source: Front Psychol. 2019 Nov 22;10:2366. doi: 10.3389/fpsyg.2019.02366 (PMC6883934; doi:10.3389/fpsyg.2019.02366)
Supplement: Supplementary file 1 [file Data_Sheet_1.docx]

**Appendix A – Search string**

ERIC and PsycINFO

( (TI (differentiat* OR adapt* OR individualiz* OR individualis* OR “ability group*” OR “achievement group*” OR “performance group*” OR grouping OR “flexible group*” OR tier* OR “mastery learn*” OR “standards based” OR “competency based” OR “proficiency based” OR “*re-teach*” OR *reteach* OR enrich* OR accelerat*) OR KW (differentiat* OR adapt* OR individualiz* OR individualis* OR “ability group*” OR “achievement group*” OR “performance group*” OR grouping OR “flexible group*” OR tier* OR “mastery learn*” OR “standards based” OR “competency based” OR “proficiency based” OR “*re-teach*” OR *reteach* OR enrich* OR accelerat*) OR AB (differentiat* OR adapt* OR individualiz* OR individualis* OR “ability group*” OR “achievement group*” OR “performance group*” OR grouping OR “flexible group*” OR tier* OR “mastery learn*” OR “standards based” OR “competency based” OR “proficiency based” OR “*re-teach*” OR *reteach* OR enrich* OR accelerat*)) AND (TI (instruct* OR teach* OR train* OR guid* OR educat* OR learn*) OR KW (instruct* OR teach* OR train* OR guid* OR educat* OR learn*) OR AB (instruct* OR teach* OR train* OR guid* OR educat* OR learn*)) AND (TI (“student* achiev*” OR “pupil* achiev*” OR “academic achiev*” OR “cognitive achiev*” OR “learn* achiev*” OR “student* perform*” OR “pupil* perform*” OR “academic perform*” OR “cognitive perform*” OR “learn* perform*” OR “student* attain*” OR “pupil* attain*” OR “academic attain*” OR “cognitive attain*” OR “learn* attain*” OR “student* improvement” OR “pupil* improvement*” OR "academic improvement*" OR “cognitive improvement*” OR “learn* improvement*” OR “student* effect*” OR “pupil* effect*” OR “academic effect*” OR “cognitive effect*” OR “learn* effect*” OR “student* learn*” OR “pupil* learn*” OR “academic learn*” OR “cognitive learn*” OR “learn* learn*” OR “student* outcome*” OR “pupil* outcome*” OR “academic outcome*” OR "cognitive outcome*” OR “learn* outcome*” OR “student* knowledge*” OR “student* skill*” OR “student* underst*” OR “learner* knowledge*” OR “learner* skill*” OR “learner* underst*” OR “pupil* knowledge*” OR “pupil* skill*” OR “pupil* underst*”) OR KW (“student* achiev*” OR “pupil* achiev*” OR “academic achiev*” OR “cognitive achiev*” OR “learn* achiev*” OR “student* perform*” OR “pupil* perform*” OR “academic perform*” OR “cognitive perform*” OR “learn* perform*” OR “student* attain*” OR “pupil* attain*” OR “academic attain*” OR “cognitive attain*” OR “learn* attain*” OR “student* improvement” OR “pupil* improvement*” OR "academic improvement*" OR “cognitive improvement*” OR “learn* improvement*” OR “student* effect*” OR “pupil* effect*” OR “academic effect*” OR “cognitive effect*” OR “learn* effect*” OR “student* learn*” OR “pupil* learn*” OR “academic learn*” OR “cognitive learn*” OR “learn* learn*” OR “student* outcome*” OR “pupil* outcome*” OR “academic outcome*” OR "cognitive outcome*” OR “learn* outcome*” OR “student* knowledge*” OR “student* skill*” OR “student* underst*” OR “learner* knowledge*” OR “learner* skill*” OR “learner* underst*” OR “pupil* knowledge*” OR “pupil* skill*” OR “pupil* underst*”) OR AB (“student* achiev*” OR “pupil* achiev*” OR “academic achiev*” OR “cognitive achiev*” OR “learn* achiev*” OR “student* perform*” OR “pupil* perform*” OR “academic perform*” OR “cognitive perform*” OR “learn* perform*” OR “student* attain*” OR “pupil* attain*” OR “academic attain*” OR “cognitive attain*” OR “learn* attain*” OR “student* improvement” OR “pupil* improvement*” OR "academic improvement*" OR “cognitive improvement*” OR “learn* improvement*” OR “student* effect*” OR “pupil* effect*” OR “academic effect*” OR “cognitive effect*” OR “learn* effect*” OR “student* learn*” OR “pupil* learn*” OR “academic learn*” OR “cognitive learn*” OR “learn* learn*” OR “student* outcome*” OR “pupil* outcome*” OR “academic outcome*” OR "cognitive outcome*” OR “learn* outcome*” OR “student* knowledge*” OR “student* skill*” OR “student* underst*” OR “learner* knowledge*” OR “learner* skill*” OR “learner* underst*” OR “pupil* knowledge*” OR “pupil* skill*” OR “pupil* underst*”)) AND (TI (secondary OR “middle school*” OR “high school*” OR “sixth-form*” OR “sixth form” OR “Key stage three” OR “Key stage four” OR “Key stage five” OR “Key stage 3” OR “Key stage 4” OR “Key stage 5” OR “junior high” OR “senior high” OR “junior college*” OR “vocational school*” OR “vocational education*”) OR KW (secondary OR “middle school*” OR “high school*” OR “sixth-form*” OR “sixth form” OR “Key stage three” OR “Key stage four” OR “Key stage five” OR “Key stage 3” OR “Key stage 4” OR “Key stage 5” OR “junior high” OR “senior high” OR “junior college*” OR “vocational school*” OR “vocational education*”) OR AB (secondary OR “middle school*” OR “high school*” OR “sixth-form*” OR “sixth form” OR “Key stage three” OR “Key stage four” OR “Key stage five” OR “Key stage 3” OR “Key stage 4” OR “Key stage 5” OR “junior high” OR “senior high” OR “junior college*” OR “vocational school*” OR “vocational education*”)) )

Web of Science

TOPIC

(differentiat* OR adapt* OR individualiz* OR individualis*) AND (instruct* OR teach* OR train* OR guid* OR educat* OR learn*) AND (“student* achiev*” OR “pupil* achiev*” OR “academic achiev*” OR “cognitive achiev*” OR “learn* achiev*” OR “student* perform*” OR “pupil* perform*” OR “academic perform*” OR “cognitive perform*” OR “learn* perform*” OR “student* attain*” OR “pupil* attain*” OR “academic attain*” OR “cognitive attain*” OR “learn* attain*” OR “student* improvement” OR “pupil* improvement*” OR "academic improvement*" OR “cognitive improvement*” OR “learn* improvement*” OR “student* effect*” OR “pupil* effect*” OR “academic effect*” OR “cognitive effect*” OR “learn* effect*” OR “student* learn*” OR “pupil* learn*” OR “academic learn*” OR “cognitive learn*” OR “learn* learn*” OR “student* outcome*” OR “pupil* outcome*” OR “academic outcome*” OR "cognitive outcome*” OR “learn* outcome*” OR “student* knowledge*” OR “student* skill*” OR “student* underst*” OR “learner* knowledge*” OR “learner* skill*” OR “learner* underst*” OR “pupil* knowledge*” OR “pupil* skill*” OR “pupil* underst*”) AND (“secondary school*” OR secondary OR “secondary education*” OR “secondary student*” OR “middle school*” Or “high school*” OR “sixth-form*” OR “Key stage three” OR “Key stage four” OR “Key stage five” OR “Key stage 3” OR “Key stage 4” OR “Key stage 5” OR “junior high” OR “senior high” OR “junior college*” OR “vocational school*” OR “vocational education*”)

**Appendix B - Variables coded in the data-extraction**

| **General**  Authors, Title, Year, Country |
| --- |
| **Design**  Study design, Number of students, Intervention (n), Control (n), Number of schools, Intervention number of schools, Control number of schools, Number of classes, Intervention number of classes, Control number of classes, Comparability control/experimental condition, Research question, Author Conclusions |
| **Intervention**  Type of differentiation, Aim of differentiation, Differentiation program, Learning environment, Selection criteria for differentiation, Instrument for discerning learning needs, What is differentiated, Executed by teacher or researcher, Degree of training or coaching for the experimental teachers, Duration of intervention, Intensity of intervention, Degree of implementation |
| **Results**  Outcomes reported for whom, Outcome variables used, Measurement instrument, Type of instrument (standardized, researcher-made, teacher-made), Effect summary, Effect size, Pretest comparability , Adjustments, Other findings, Drop-outs |
| **Student-level variables**  Student level, Student age (mean, range) , Student ethnicity, Student socio-economic background,  Other student characteristics |
| **Class-level variables**  Grade, Subject (topic), Classroom composition (diversity students), Class-size |
| **Teacher-level variables**  Teacher age, Teacher gender, Teacher education, Teacher knowledge / beliefs / attitudes / motivation about differentiation, Other teacher characteristics |
| **School-level variables**  School size, School type, School policy for differentiation (i.e. time for professionalization, supportive school leader, facilities), Collaboration among teachers |

**Appendix C - Flow Chart**


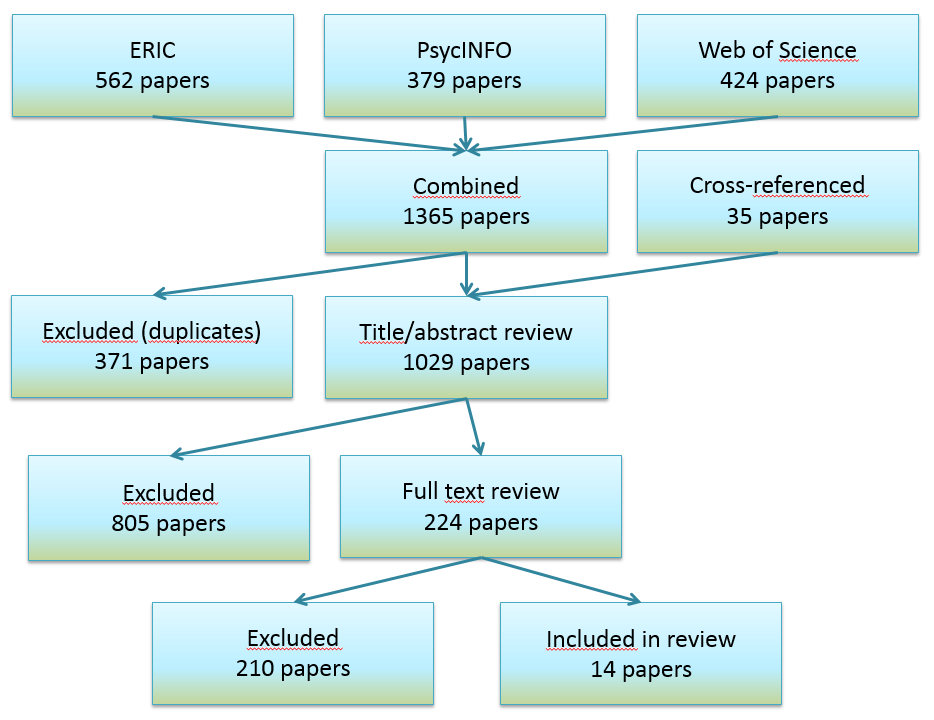


*Figure 1.* Flow chart of the selection process.

Note. Although 14 papers were selected, there were two instances in which two papers reported on the same project. After merging these papers, in the end **12 unique studies** were included in the review study.

**Appendix D – Forest Plots of Study Effects**


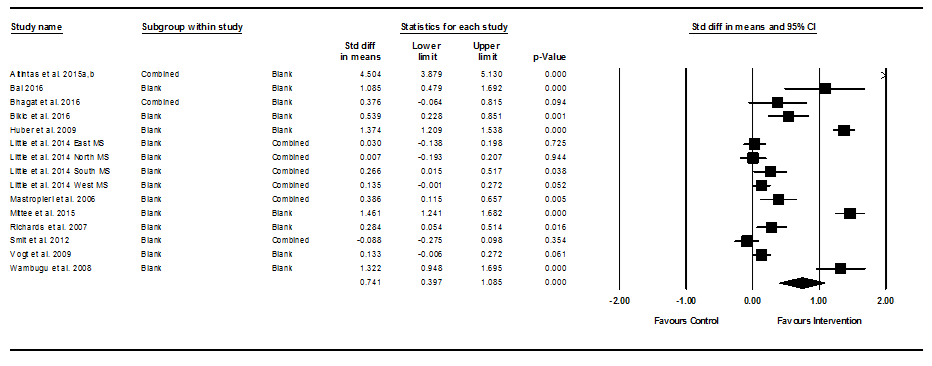


*Figure 2*. Forest plot of all of the included studies. Squares represent average effects of individual studies and the diamond represents a summary effect of within class differentiation on student achievement across all studies. The horizontal lines represent confidence intervals.


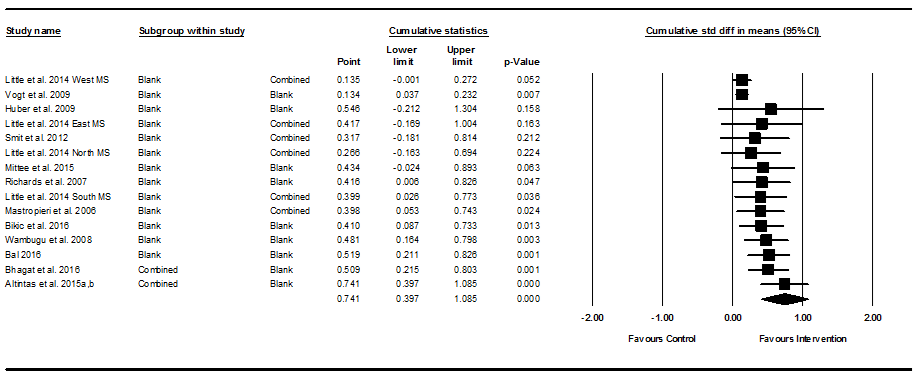


*Figure 3*. Forest plot of the cumulative analysis. The point estimate of each line represents the combined effect of that study and the previous study/studies. Therefore the cumulative analysis shows how much the effect size shifts when studies are added.

Note: Studies are ordered relative to their standard errors, starting from relatively large studies (small SE) to relatively small studies (large SE).


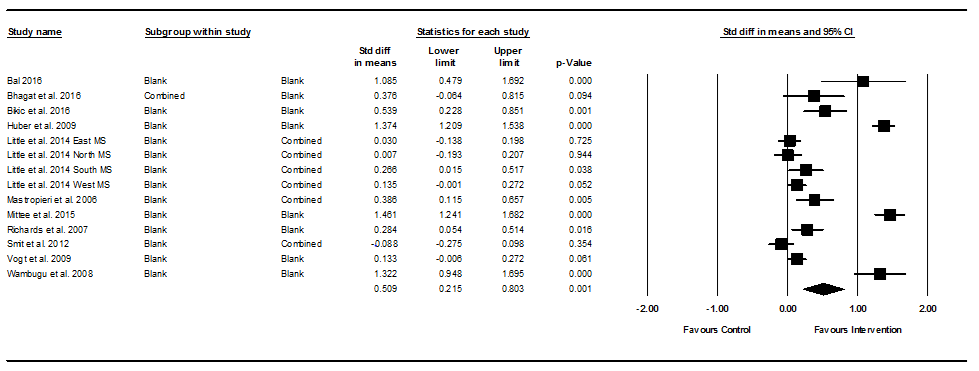


*Figure 4.* Forest plot of included studies, excluding one outlier. Squares represent average effects of the individual studies and the diamond represents a summary effect of within class differentiation on student achievement across the studies. The horizontal lines around the squares and the diamond represent confidence intervals.

**Appendix E – Funnel Plot**


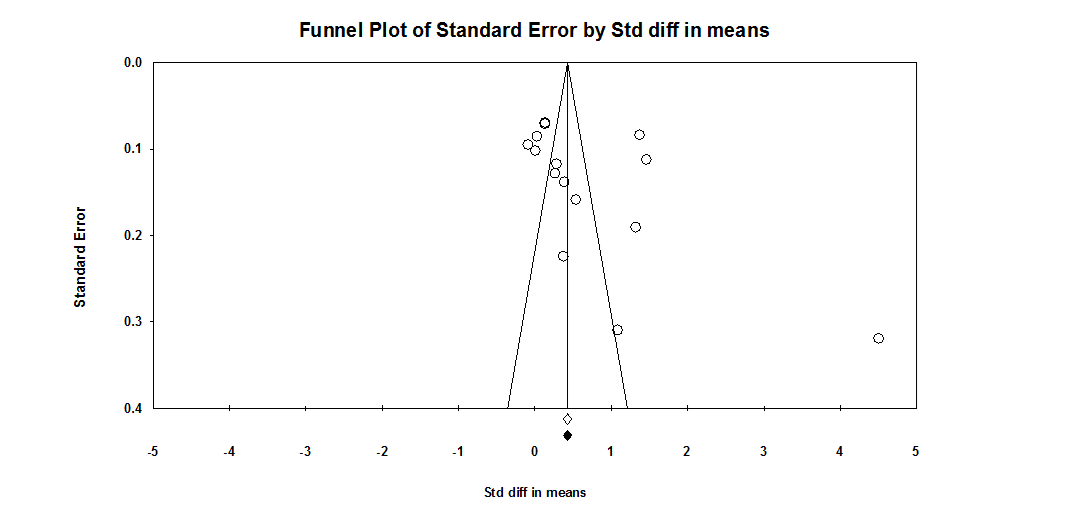


*Figure 5.* Funnel plot of the included studies. The white diamond indicates the summary effect of within class differentiation on student achievement across all studies. Duvall and Tweedie’s trim and fill approach was used to estimate missing values. The black diamond indicates the point estimate adjusted for potential publication bias (under the random effects model) which is in this case equal to the point estimate of our effect determined across studies.

**Appendix F – Suggested reporting elements in within-class differentiation studies**

| **Reporting element** | **Examples** |
| --- | --- |
| What was differentiated? | Content, process, product, time, instructions, support, material, learning environment |
| How was the differentiation organized? | Flexible ability groups, peer-coaching, tiered content |
| Which information was used to gain insight in students’ learning needs? | Formative tests, student preferences, observations |
| How was the intervention planned, executed and evaluated? | The procedure before, during, and after the lesson used to successfully execute the approach |
| What was the goal of the differentiation intervention? | Improving the achievement of weaker learners, excelling faster learners, increasing overall class achievement |
| Was the intervention (mainly) teacher directed or was an ICT application used? | Did the teacher (or researcher) direct the differentiated instruction or was the approach managed by an ICT application? |
| What was the duration and intensity of the intervention | Duration in weeks, number of lessons / hours per week |
| Which type of support did teachers need to execute the intervention? | Workshops, coaching, development of materials, feedback |
| What did the intervention cost? | Time, knowledge, and resources needed to adopt the innovation |
| Was the intervention congruent to teachers’ daily practice? | A reflection on the question to which degree the intervention fits the circumstances in which teachers work |
| Implementation | Was the intervention implemented well? How was the implementation of differentiation measured? Which processes facilitated or hampered the implementation? What are suggestions for implementation in practice? |
| Context | Information on context factors relevant to the intervention e.g. teacher knowledge, experience, classroom characteristics |
| Outcomes | What was the effect of the intervention on (different types of) student outcomes? Were there differential effects of the intervention for students of varying backgrounds (e.g. low, average and high ability students)? Were there other factors that could have caused the effect? |
